# Near real-time forecasting for cholera decision making in Haiti after Hurricane Matthew

**DOI:** 10.1371/journal.pcbi.1006127

**Published:** 2018-05-16

**Authors:** Damiano Pasetto, Flavio Finger, Anton Camacho, Francesco Grandesso, Sandra Cohuet, Joseph C. Lemaitre, Andrew S. Azman, Francisco J. Luquero, Enrico Bertuzzo, Andrea Rinaldo

**Affiliations:** 1 Laboratory of Ecohydrology, School of Architecture, Civil and Environmental Engineering, École Polytechnique Fédérale de Lausanne, Lausanne, Switzerland; 2 Centre for the Mathematical Modelling of Infectious Diseases, Department of Infectious Disease Epidemiology, London School of Hygiene & Tropical Medicine, London, United Kingdom; 3 Epicentre, Paris, France; 4 Department of Epidemiology, Johns Hopkins University Bloomberg School of Public Health, Baltimore, Maryland, United States of America; 5 Epicentre, Geneva, Switzerland; 6 Department of International Health, Johns Hopkins University Bloomberg School of Public Health, Baltimore, Maryland, United States of America; 7 Department of Environmental Sciences, Informatics and Statistics, University Ca’ Foscari Venezia, Venezia Mestre, Italy; 8 Department of Civil, Environmental and Architectural Engineering, University of Padua, Padova, Italy; The Pennsylvania State University, UNITED STATES

## Abstract

Computational models of cholera transmission can provide objective insights into the course of an ongoing epidemic and aid decision making on allocation of health care resources. However, models are typically designed, calibrated and interpreted *post-hoc*. Here, we report the efforts of a team from academia, field research and humanitarian organizations to model in near real-time the Haitian cholera outbreak after Hurricane Matthew in October 2016, to assess risk and to quantitatively estimate the efficacy of a then ongoing vaccination campaign. A rainfall-driven, spatially-explicit meta-community model of cholera transmission was coupled to a data assimilation scheme for computing short-term projections of the epidemic in near real-time. The model was used to forecast cholera incidence for the months after the passage of the hurricane (October-December 2016) and to predict the impact of a planned oral cholera vaccination campaign. Our first projection, from October 29 to December 31, predicted the highest incidence in the departments of Grande Anse and Sud, accounting for about 45% of the total cases in Haiti. The projection included a second peak in cholera incidence in early December largely driven by heavy rainfall forecasts, confirming the urgency for rapid intervention. A second projection (from November 12 to December 31) used updated rainfall forecasts to estimate that 835 cases would be averted by vaccinations in Grande Anse (90% Prediction Interval [PI] 476-1284) and 995 in Sud (90% PI 508-2043). The experience gained by this modeling effort shows that state-of-the-art computational modeling and data-assimilation methods can produce informative near real-time projections of cholera incidence. Collaboration among modelers and field epidemiologists is indispensable to gain fast access to field data and to translate model results into operational recommendations for emergency management during an outbreak. Future efforts should thus draw together multi-disciplinary teams to ensure model outputs are appropriately based, interpreted and communicated.

## Author summary

Mathematical models of cholera transmission can help predict the dynamics of outbreaks in near real-time in order to inform decision making for emergency management. Following the passage of Hurricane Matthew on cholera-struck Haiti in October 2016, a large oral cholera vaccine campaign targeting approximately 760,000 individuals was planned to minimize the risk of cholera transmission after the heavy hurricane rainfall. We used a reliable spatially-explicit mathematical model and state-of-the-art data assimilation techniques to predict the number of averted cases owing to the vaccination campaign. We accounted for different forecasts of precipitation patterns, a well known risk factor for the amplification of cholera epidemics, and reported near real-time projections of cholera cases for November and December 2016 to a group of epidemiologists and field researchers of Médecins Sans Frontières. Model results were then translated into operational recommendations during the outbreak management. Our projections highlighted that the departments of Grande Anse and Sud were at risk of a second epidemic wave, thus supporting the planned vaccination campaign therein. Our projections provided estimates and prediction intervals of the actual number of averted cases due to OCV per each of the 140 Haitian communes.

## Introduction

A major cholera epidemic has ravaged Haiti since October 2010 with more than 800,000 reported cases and close to 10,000 reported deaths as of December 2017 (http://mspp.gouv.ht). On October 4-5, 2016 Hurricane Matthew struck Haiti, causing flooding and infrastructure damage in the historically less cholera-affected South-West of the country [1]. The drastic worsening of sanitary conditions [2–4] and the reduced accessibility to safe water caused by the devastating winds and the heavy rainfall brought by Matthew created favorable conditions for the rapid transmission of cholera. In particular, such conditions developed in the departments of Sud and Grande Anse, where preexisting immunity against cholera was thought to be much lower than elsewhere in Haiti [2]. Humanitarian organizations supporting the Haitian Ministry of Health (Ministère de la Santé Publique et de la Population, MSPP) rapidly responded in the region as an increase of suspected cholera cases was reported (e.g., [5]). Given the limited resources to respond to the epidemic, quick decisions needed to be made on how and where to intervene to mitigate the threat of a large cholera outbreak. Intervention strategies included a collaboration among 30 partners to support DINEPA (Direction National de l’Eau Potable et de l’Assainissment) in providing safe water, sanitation and hygiene promotion in shelters, residential centers and medical facilities of 40 communes [6], mainly through WASH campaigns. In parallel to these activities, MSPP planned a large vaccination campaign in the communes most affected by the hurricane.

A collaboration between a humanitarian organization on the field, Médecins Sans Frontières (MSF), and an academic group based at Ecole Polytechinque Fédérale de Lausanne (EPFL) and experienced in modeling the Haitian cholera outbreak [7–15] allowed for the rapid development of a spatio-temporal cholera forecasting model capable of assimilating data coming in from the field on a regular basis to answer important operational questions. This collaborative effort had two main objectives. First, we wanted to assess the likelihood of a large cholera outbreak, including the probability of a second seasonal peak. Second, conditional on the assessment of imminent cholera risk, we wanted to better understand the potential impact (e.g., cases/deaths averted) of a planned targeted oral cholera vaccine campaign in hurricane-affected areas.

Cholera is a waterborne disease caused by the bacteria *Vibrio Cholerae*, whose main symptoms are severe acute watery diarrhea and vomiting leading to dehydration, which progresses to hypovolemic shock and death if not treated. *V. Cholerae* shed by infected individuals might survive for several days in the environment and it may even thrive in fresh water [16]. Rainfall seasons and flooding events play an important role in water contamination, by fostering bacterial dispersal from open air defecation sites and other untreated human waste to the local watersheds, thus enhancing the water-to-human transmission of the disease.

Several studies in literature have found a correlation between climatic conditions and cholera transmission [15]. Most countries where associations between rainfall and cholera risk have been studied experience endemic disease transmission [17–20]. Empirical studies have shown a range of correlations, both positive and negative, endowed with time lags ranging from weeks to months. For instance, rainfall has been found to enhance cholera transmission in dry regions, while it can buffer the propagation of the disease in wet regions due to a dilution effect [17–20]. Such variability may reflect the variety of potential mechanisms whereby rainfall may alter infection risk, specifically: through flooding, leading to raw sewage contamination of water sources [17, 18] and enhanced exposure to human-to-human transmission due to crowding [21]; increased rainfall-driven iron availability in environmental waters that enhances pathogen survival and expression of toxins [22]; and decreased water levels leading to increased usage of unsafe water sources [23, 24]. A clear correlation between rainfall and enhanced transmission is found also in regions hit by cholera epidemics [21, 24, 25]. Notably, for the Haiti outbreak, this link has been found empirically [2] and justified theoretically [3, 4] at all spatial scales and locations examined. Intense rainfall events were significantly correlated with increased cholera incidence with lags of the order of a few days, and forcing dynamic models with rainfall data invariably resulted in improved fits of reported infection cases.

As demonstrated by previous models of cholera transmission in Haiti, both rainfall and baseline immunity to cholera play an important role in shaping the performance of models in reproducing measured incidence data [2–4], posing challenges to real-time inference relevant to decision making.

Our main interest, at the time the vaccination was planned, was to understand how likely the risk of a marked increase in cholera cases was, with a particular focus on the areas most affected by the hurricane. Secondly, we aimed at forecasting the impact of the vaccination campaign in terms of averted cases, an endeavour only possible via robust modelling. Both of these heavily depended on forecasts of future rainfall. Although the Haitian cholera model has been developed for several years [3, 8, 14, 26], it underwent considerable evolution during the Matthew emergency in order to provide real-time projections. The results presented here are based on the latest version of this model to provide a coherent framework among the different projections computed during the emergency and additional analysis. We refer to [27] and [28] for the original reports published at the time during the Hurricane. Here, we provide details on the updated dynamical model of cholera transmission and its performance in computing near real-time assessments of cholera risk and potential impact of vaccination in the Haitian context. We provide insights on how the model, using data assimilation (DA) approaches, can be further optimized to provide rapid and field-relevant data for cholera control decision making in other settings. Finally, we discuss the importance of collaborative efforts to collect and share key data in real-time and use them in appropriately designed models to provide data-driven guidance for public health decision makers in cholera epidemics.

## Results

### Cholera dynamics after Hurricane Matthew

**Fig 1:**
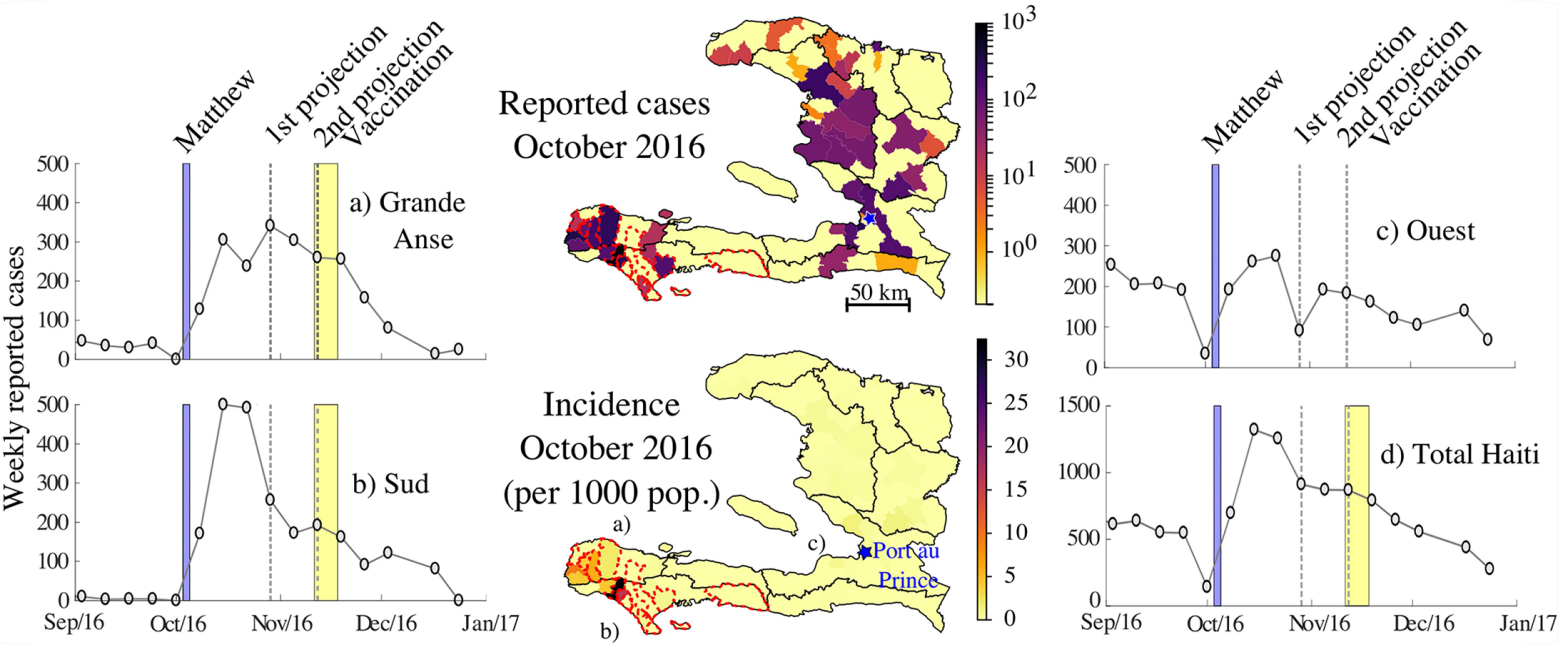
Reported cases after Matthew. Maps of the reported cholera cases and the associated incidence during October 2016 at the communal level with the detailed weekly dynamics for the departments most affected after Matthew (Grande Anse (a), Sud (b) and Ouest (c)), and for the whole country (panel d).

When Hurricane Matthew struck Haiti on October 4, the average daily rainfall peaked at over 500 mm/d (S1 Fig), especially in the coastal departments of Grande Anse and Sud, which reported the majority of hurricane-related damages [29]. In these two departments, suspected cholera incidence rapidly exploded from a few reported cases per week to almost 500 cases per week (week 43), with a total of 1349 and 1533 reported cases for Grande Anse and Sud, respectively, between October 5 and November 6 (Fig 1) [30]. Cholera incidence also increased in the capital Port-au-Prince (Ouest department), with 438 cases reported over the same period.

The main events regarding the OCV planning and the modeling efforts followed this time-line:

- October 4-5: Hurricane Matthew struck Haiti;
- October 10: the Global Task Force for Cholera Control Oral Cholera Vaccine Working Group approved the request made by the MSPP for 1 million doses of OCV in order to vaccinate areas affected by the Hurricane Matthew [31];
- October 15: epidemiological peak at the national level (1507 weekly cases);
- October 18: modeling collaboration started;
- October 25: MSPP decision on how to distribute the 1 million doses of OCV;
- November 11: first modeling report describing cholera projection from October 29 to December 31 [27];
- November 11-18: OCV campaign targeting 760,000 persons in Grande Anse and Sud.
- December 23: second modeling report assessing the impact of the OCV campaign on cholera forecasts for the period from November 12 up to December 31 [28].

In this emergency situation, the results were not yet ready when the vaccination campaign was planned, and the communes selected for the vaccination were decided by the Ministry of Health and partners involved in the campaign independently of the model results. The modeling results for the first projection were issued in a report at the beginning of the vaccination campaign. The second projection was issued some weeks after the end of the campaign. Those findings were useful to corroborate the expected impact of the vaccination campaign, in particular the fact that departments targeted in the planned vaccination campaign (Grande Anse and Sud, the mostly affected by the hurricane) were the departments with the higher risk of an increase in cholera cases during the month of December. Model results in other departments which had been historically more at risk of cholera (such as Nord) or more populated (such Ouest and Center) showed a lower risk for December 2016.

### Model setting for cholera projections

We calibrated an ensemble of *N* = 1000 trajectories of the spatially-distributed SIRB model using cholera incidence data at the communal level from week 7 (February 6, 2016) to week 45 (October 28) through a data augmentation procedure (see Materials and methods and S1 Appendix for model details, and S2 Appendix for details on DA). Satellite-based rainfall estimates, obtained from the Global Precipitation Mission (GPM) [32], were the main environmental drivers for the model during this period, while departmental epidemiological reports from the beginning of the epidemic (2010) to the start of the calibration period (February 6, 2016) were used to compute the initial number of immune individuals and the initial bacterial concentration in each department (S1 Appendix). DA is particularly well suited for operational forecasts, i.e., for the assimilation of the newly available data in near real-time and the subsequent computation of cholera projections avoiding the repetition of costly calibration approaches.

Precipitation forecasts from the Climate Forecast System (CFS) were used as drivers to produce two cholera projections, the first from week 45 (October 29), the second from week 47 (November 12), to the end of 2016. Three scenarios of rainfall forecasts were considered for both projections:

S1 Each model realization was forced by a precipitation trajectory assigned by bootstrapping from the last 24 CSF forecasts computed in the week before the beginning of the projections;
S2 A heavy rainfall scenario was assigned to all model trajectories by selecting, among the 24 CSF forecasts of S1, the forecast with the maximum total precipitation over Haiti during the forecast period. This set-up aims at representing the worst-case scenario;
S3 We used the actual precipitation data (as measured *a posteriori* by GPM) to retrospectively compare model forecasts without including uncertainty in rainfall.

For each scenario, results were computed with and without vaccination campaign, which consisted in the administration of around 760,000 OCV doses in nine communes of Grande Anse and seven communes of Sud from November 11 to November 18, 2016 (the specific communes are detailed in S1 Appendix). Vaccine efficacy and coverage are the model parameters that determine the impact of the vaccination campaign during the forecast. As their values cannot be determined prior to the vaccination, different forecast scenarios can be produced by varying these parameters. Here we present the forecasts associated with a vaccine efficacy (*η*) of 87% and a one-dose vaccine coverage (*ξ*) of 90%, consistently with evidence from a number of recent clinical trials and vaccine efficacy/effectiveness studies [12, 33, 34]. After the forecast, a sensitivity analysis to these parameters was carried on to asses to reliability of this assumption.

### First projection: Epidemic evolution and vaccine impact after hurricane passage (October 29, 2016)

**Fig 2:**
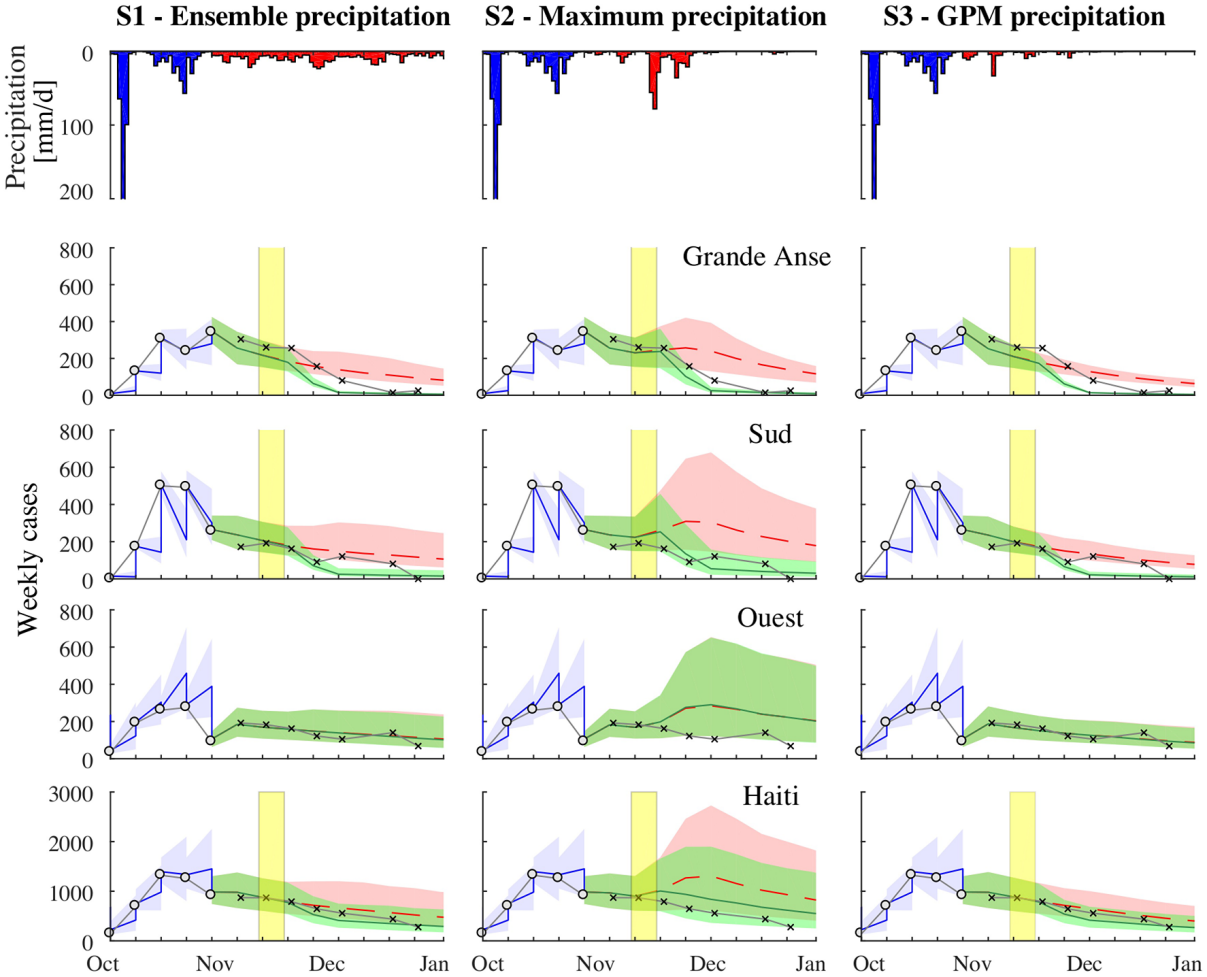
First cholera projection. From September 3 to October 29, 2016: comparison between the weekly reported cases (white circles) and the model results forced by the DA procedure in the departments of Grande Anse, Sud, Ouest, and over all Haiti. The median of the ensemble is the blue line, while the blue area represents the ensemble 0.05-0.95 confidence interval. From October 29 to December 31, 2016: comparison between the weekly reported cases (crosses) and the model forecast (the ensemble median is the dashed line, the area represents the ensemble 0.05-0.95 confidence interval). Results in green are obtained considering the OCV campaign with vaccine efficacy set to *η* = 87% and coverage *ξ* = 90%. Results without OCV campaign are in red. The yellow shaded area shows the timing of the OCV campaign. Panels in the first row depict the average measured precipitation (blue) and the forecasted precipitation (red) under scenario S1 (left), S2 (center), and S3 (right).

Our first projection in this epidemic considered the potential evolution of the outbreak and vaccine impact from October 29 to December 31, 2016 (Fig 2). During this period, we estimated an average of 7274, 10384 and 6955 cases across Haiti in scenarios S1, S2 and S3, respectively. Under all scenarios of forecasted rainfall, two departments, Grande Anse and Sud, consistently had the highest cumulative incidence and accounted for the 47% (90% PI 41-54%) of the total cases during the projection in S1, 45% (90% PI 38-51%) in S2, and 45% (90% PI 39-51%) in S3. Within these two departments and Ouest, which has the largest population in Haiti and accounted for the 19% (90% PI 16-21%) of the total projected cases in S1, the forecasts suggested that incidence was likely to reach a peak and then start decreasing in the first two weeks of November (Fig 2).

Rainfall scenarios greatly influenced the probability of a second cholera peak occurring at the beginning of December in Grande Anse, Sud and Ouest departments (Fig 2). In the maximum rainfall scenario (S2), where heavy rains were expected in late November, the risk for a further increase of weekly cases during the projection was 5% in Grande Anse and 70% in Sud (see Table S4.1, S4 Appendix), the difference mainly due to a larger initial number of cases at the beginning of the projection in Grande Anse. In fact, for both Grande Anse and Sud, the attack rate of the outbreak during the second half of November and December (weeks 46-51) was forecasted to be of similar intensity of the attack rate in the first six weeks of the outbreak (week 40-45), with median values passing from 32 to 27 cases per 10,000 persons in Grande Anse and from 24 to 20 cases per 10,000 persons in Sud (see Table S4.3, S4 Appendix). In the ensemble rainfall scenario (S1), considering the ensemble of precipitation forecasts for November and December, the probability of an increase in cholera cases was 0.6% in Grande Anse and 15.6% in Sud. When considering the rainfall that actually occurred (S3, which was not possible prospectively), the increase risk was significantly lower than the other scenarios, with a 0.1% risk in Grande Anse and a 6% risk in Sud (see Table S4.1, S4 Appendix).

On October 25, 2016, the Haitian MSPP decided to vaccinate several communes of the departments of Sud and Grande Anse, mainly selected based on hurricane damages, reported cholera cases and expert opinion. Vaccination started shortly after, on November 11.

The simulated OCV campaign halved the number of cases during the first six weeks (weeks 46-51) in both Sud and Grande Anse, where vaccination activities were assumed to occur, while there was little to no impact elsewhere in the country (Tables S4.2-S4.4, S4 Appendix). The impact of the OCV campaign in terms of averted cases up to December 31 was greatest under the scenarios presenting the largest risk of a second cholera peak. In fact, in the maximum rainfall scenario (S2), we found that a median of 1830 cases (90% PI: 1002-3432 cases) would be averted, whereas 1104 (90% PI: 758-2044 cases) and 943 (90% PI: 733-1394) were expected in the ensemble (S1) and actual rainfall (S3) scenarios (see Table 1 for the cases averted by department). After inclusion of OCV campaigns in the models, the forecasted epidemic curves decreased in the areas targeted with OCV, slightly underestimating the reported cases in S1 and S2 (Fig 2).

**Table 1:** Cases averted. Projections of the cases averted (median and 90% prediction intervals) from the beginning of the OCV campaign up to December 31 in Grande Anse and Sud.

Scenario	First projection		Second projection	
	Grande Anse	Sud	Grande Anse	Sud
S1	525 (371-901)	547 (361-1079)	739 (493-1265)	865 (520-1492)
S2	835 (476-1284)	995 (508-2043)	930 (572-1546)	1139 (602-1930)
S3	459 (345-579)	462 (344-683)	682 (511-976)	721 (516-1071)

**Fig 3:**
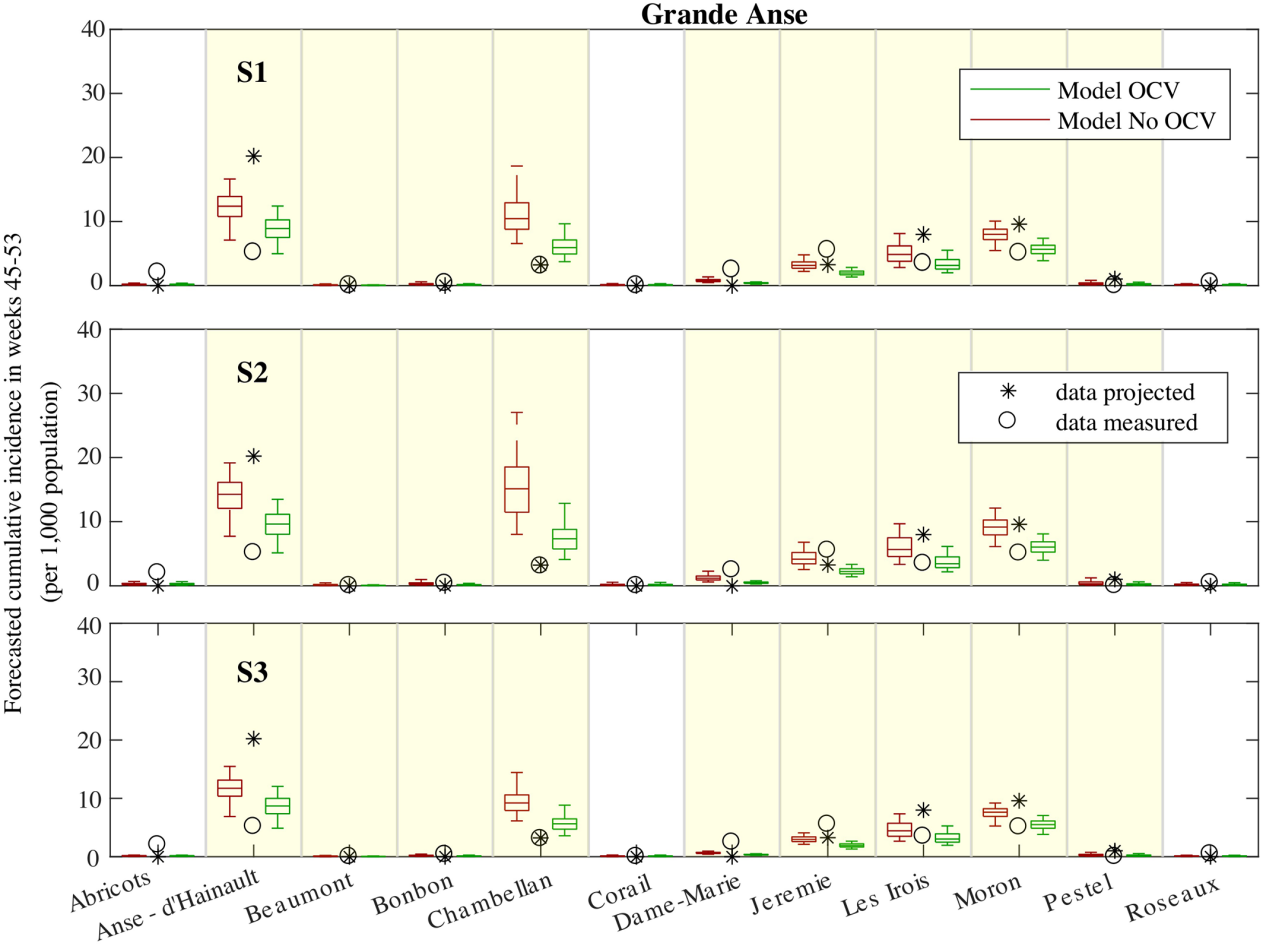
Forecasted incidence. Box-plot (percentiles 0.05, 0.25, 0.5, 0.75, 0.95) of the cumulative incidence (total number of new cases divided by the population size) forecasted in each commune of Grande Anse after the vaccination period (from November 12 to December 31, weeks 45-53) during the first projection. Results are obtained with (green) and without (red) the deployment of the OCV campaign in the communes highlighted in yellow. The cholera projections are driven by an ensemble of forecasted rainfall (S1), the maximum forecasted rainfall (S2), and the actual rainfall (S3). The circle represents the measured incidence during the same period, while asterisks are a constant projection based on the cases reported from the occurrence of Matthew up to the projection date (from October 4 to October 29).

**Fig 4:**
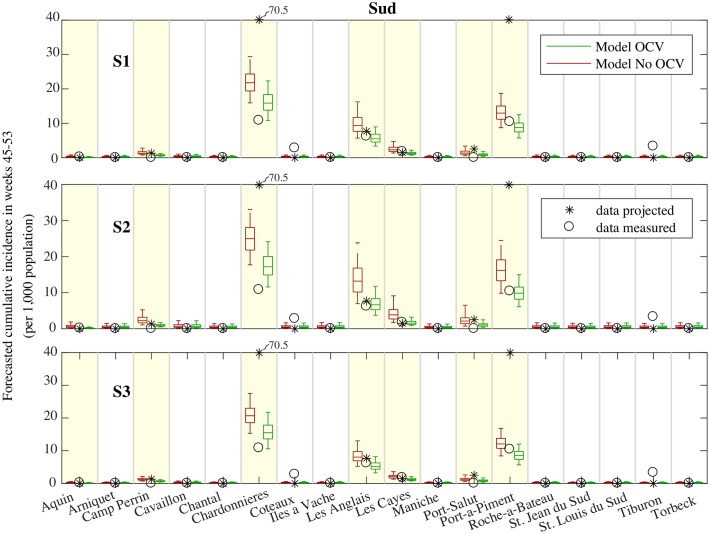
Forecasted incidence. Same as Fig 3 for the communes in Sud.

The cumulative incidence at the communal level(Fig 3 for Grande Anse and Fig 4 for Sud) shows that all the communes reporting the largest incidence in the first weeks after Matthew were included in the OCV campaign (see asterisk in Figs 3 and 4), e.g. Anse-D’Hainault, Moron and Les’Irois in Grande Anse, and Chardonnieres and Port-a-Piment in Sud. These communes still recorded the largest incidence after the vaccination campaign (circles in Figs 3 and 4). Model forecasts without OCV indicate a large risk for these communes, in general overestimating the occurred incidence (especially for scenario S3). The inclusion of the OCV campaign reduces the forecasted cumulative incidence, with results that are closer to the reported data. Note, however, that the OCV forecasts underestimated the incidence in Jeremie (Grande Anse), which is the most populated commune in the department.

OCV was implemented also in a number of other communes having a low recorded incidence after Matthew to limit the possible spread of the disease. The forecasted incidence for these communes was low and results were not impacted by the OCV campaign.

Model forecast showed a low risk also for the remaining communes which were not interested by the OCV campaign. However, some of these communes recorded an increased incidence during the projection period (e.g. Abricots in Grande Anse, and Coteaux, Tiburon in Sud).

The detailed forecasted and averted cases by commune are reported in Tables S4.9 and S4.10 in S4 Appendix.

### Second projection: Time of vaccine deployment (12-November-2016)

We updated our projections on November 12, assimilating the newly available epidemiological and rainfall data, to provide up-to-date forecasts of epidemic evolution and vaccine impact.

During the time period between the two projections, the number of weekly reported cases slightly decreased in Grande Anse (from 341 to 260 cases) and maintained almost constant in Sud (from 256 to 292 cases), while the updated ensemble of forecasted rainfalls led to a lower daily maximum, from 78 mm/d to 33 mm/d.

**Fig 5:**
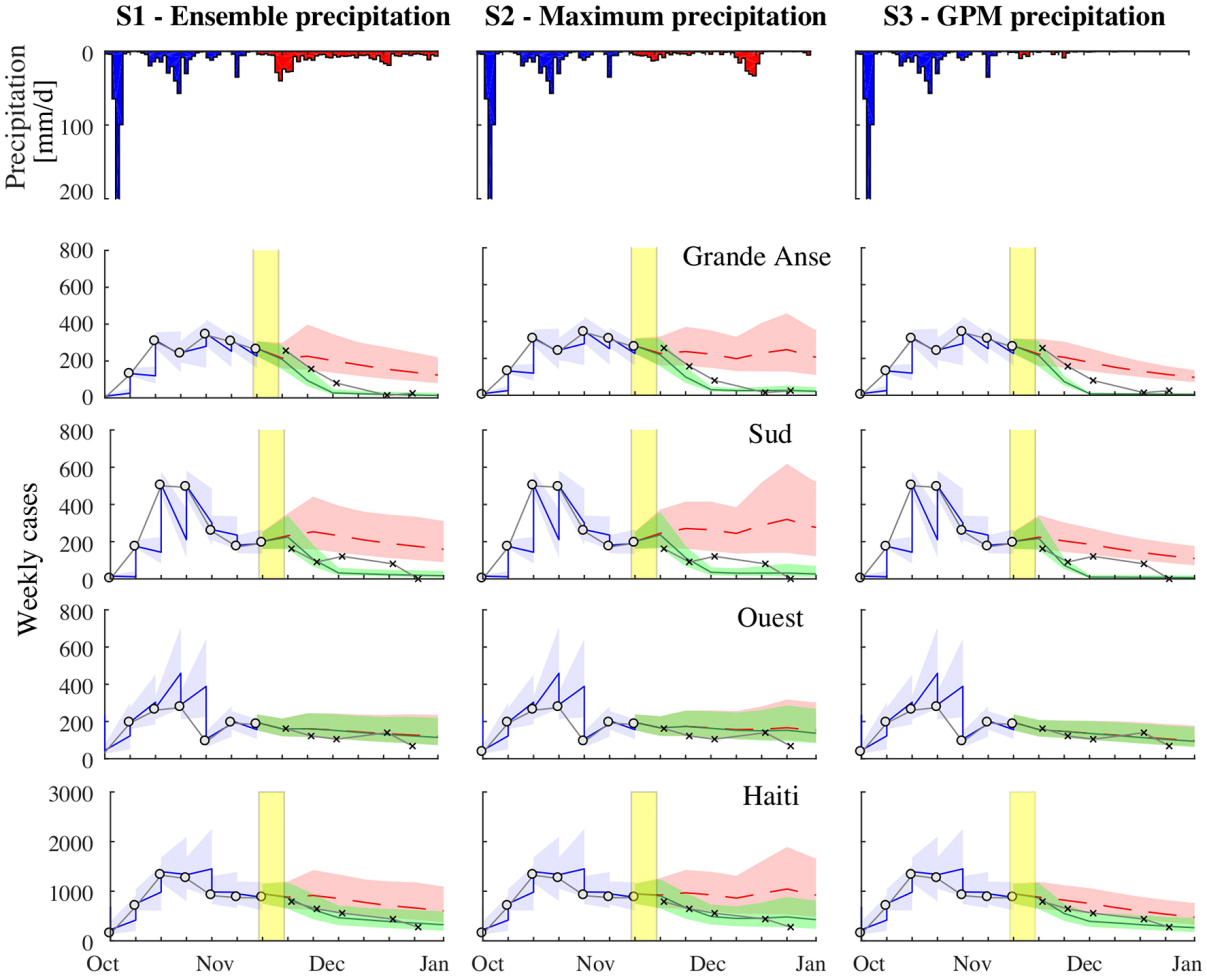
Second cholera projection. From September 3 to November 12, 2016: comparison between the weekly reported cases (white dots) and the model results forced by the DA procedure in the departments of Grande Anse, Sud, Ouest, and over all Haiti. The median of the ensemble is the blue line, while the blue area represents the ensemble 0.05-0.95 confidence interval. From November 12 to December 31, 2016: comparison between the weekly reported cases (crosses) and the model forecast (the ensemble median is the dashed line, the area represents the ensemble 0.05-0.95 confidence interval). Results in green are obtained considering the OCV campaign with vaccine efficacy set to *η* = 87% and coverage *ξ* = 90%. Results without OCV campaign are in red. The yellow shaded area shows the timing of the OCV campaign. Panels in the first row depict the average measured precipitation (blue) and the forecasted precipitation (red) under scenario S1 (left), S2 (center), and S3 (right).

In the second projection (Fig 5), the ensemble rainfall scenario forecasts S1 suggested slightly slower decreases in incidence compared to the first projection and the maximum rainfall scenario forecasts S2 still suggested a second peak, although closer to the end of the year and of reduced magnitude. In both scenarios, the attack rate during weeks 46-51 was non-negligible in both Grande Anse and Sud: 24 cases (90% PI 17-39) per 10,000 persons in Grande Anse and 17 cases (90% PI 11-28) in Sud in the ensemble rainfall scenario S1. Similarly, in the maximum rainfall scenario S2, the attack rates were 29 (90% PI 19-47) per 10,0000 persons in Grande Anse and 21 cases (90% PI 12-35) in Sud (see Tables S4.6, S4.7 in S4 Appendix).

In this second projection, the estimated number of cases averted by OCV campaigns was greater than in the first projection in both Sud and Grande Anse (Table 1). In the maximum rainfall scenario forecasts S2, 2192 (90% PI 1237-3638) cases were averted with vaccination country-wide with most of the averted cases in Sud and Grande Anse (see Table S4.2 S4 Appendix). Like the first projections, the forecasts with OCV fit the observed epidemic curves better in Sud and Grande Anse than forecasts without OCV (Fig 5).

### Sensitivity to coverage and vaccine efficacy

While our primary forecasts of vaccine impact relied on vaccine efficacy estimates from the literature *η* = 0.87 [12]) and assumptions about a large vaccination coverage (*ξ* = 0.9), here we wanted to look for the optimal values of these quantities to reproduce the reported cholera cases after the vaccination with the minimum error. To this goal, the cholera second projection considering the measured rainfall (scenario S3) is tested under different values of *η* and *ξ*, and the results are compared in terms of the ensemble Root Mean Squared Error (RMSE) with respect to the reported cases.

**Fig 6:**
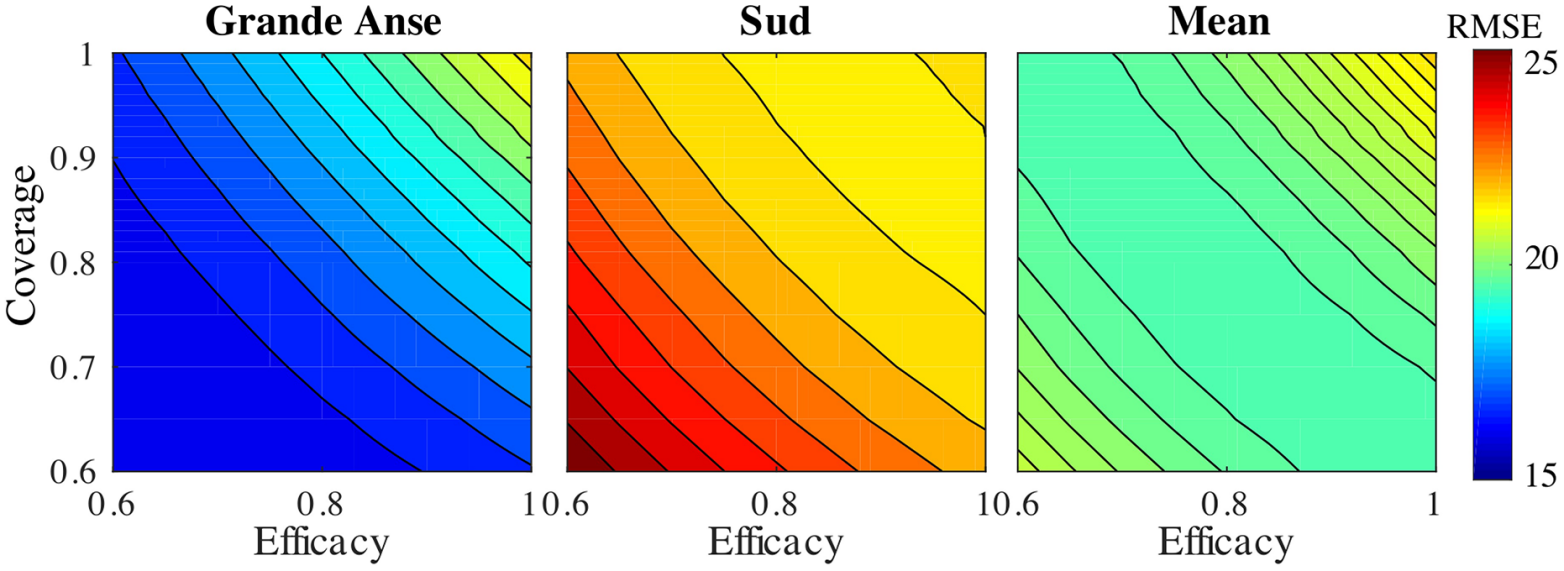
Sensitivity of the ensemble RMSE to the OCV coverage and the vaccine efficacy during the second projection using measured rainfall (scenario S3). The minimum mean RMSE is obtained for coverage *ξ* = 0.6 and efficacy *η* = 1.0 (RMSE = 19.46), however the structure of the error is almost symmetric (RMSE = 19.49 for *ξ* = 1.0 and efficacy *η* = 0.6) indicating identifiability issues between coverage and efficacy. A slightly higher error, RMSE = 19.98, is associated to the second projection (*ξ* = 0.9 and efficacy *η* = 0.87).

Fig 6 shows the ensemble RMSE in the communes targeted by the OCV campaign in Grande Anse and Sud, and their average as a function of vaccine efficacy and coverage. Combinations of such parameters that minimize the error allows an approximate estimate of the actual efficacy and coverage of the implemented OCV campaign. The larger model errors in Sud are mainly due to a slight increase in the number of cases in the first two weeks after the vaccination, dynamic that the model is not able to capture (see Fig 5). We can see that the area with the lowest errors in all departments is obtained from a combination of relatively large efficacy and coverage, thus suggesting a good performance of the OCV campaign. However the error increases when both efficacy and coverage quantities are close to 1. Considering that the estimated coverage for this campaign is about 90% [35], the value of the vaccine efficacy that minimizes the mean error is about *η* = 0.7, smaller than the one used during the projections (*η* = 0.87 [12]). Additional long-term data should be considered for a better evaluation of the vaccine efficacy.

## Discussion

This study presents a first attempt at a multi-disciplinary effort to compute near real-time projections of an ongoing cholera epidemic and to translate model outputs into decision-making to manage emergencies in the field. The close collaboration established with field epidemiologists allowed modelers to directly access data in near real-time, at a spatial resolution that was not available in previous similar efforts. In fact, previous cholera modeling and projection attempts in Haiti through SIRB-based models were mainly calibrated on departmental-level data released by the Haitian MSPP [3, 26, 36], or aggregations of data at different scales [4]. In these cases, SIRB models could retrieve the general trends of the epidemic and produce reliable long-term projections of the Haitian epidemics, say to evaluate incidences immediately following the beginning of the emergency [36], or to assess the probability of extinction of the epidemic [26]. Here, the weekly reported communal cases during the Matthew’s emergency and their assimilation through a DA scheme, the ensemble Kalman filter, radically improved the local accuracy of the model projections by sequentially updating the state variables and the key parameters of the model. This strategy resulted more effective than classic calibrations, e.g. through Monte Carlo Markov Chain on long time series, especially for short term forecasts (see [14]).

The first cholera projection, from October 29 (Fig 3) up to December 31 2016, provided a quantitative estimation of the spatial and temporal features of the cholera outbreak and their uncertainty expressed in terms of 90% predictive intervals. The highest cumulative incidence was predicted to occur in the departments of Grande Anse and Sud, which proved the most damaged after the hurricane. Moreover, under the worst scenario (the model driven by the maximum rainfall forecast, scenario S2) a second wave of infections was foreseen to peak at the beginning of December. The peak was foreseen to hold a median attack rate of 27 cases per 10,000 persons in Grande Anse (90% PI 15-41) and of 20 in Sud (90% PI 10-42). The above figures are comparable to the intensity recorded during the first four weeks of the outbreak (32 in Grande Anse, 90% PI 29-36, and 24 in Sud, 90% PI 22-27, see Table S4.3 in S4 Appendix). These model results reinforced the fear for the potential role of upcoming heavy rainfall events in revamping the epidemic. From an operational viewpoint, such projections corroborated the expert’s opinion and encouraged the implementation of the planned OCV campaign that started on November 11 in these departments.

MSF provided detailed information about the spatio-temporal allocation of the OCV doses (see S1 Appendix) at the beginning of the campaign. This enabled a near real-time assessment of the short-term impact of the intervention strategy, using only the available information about the reported cases and precipitation forecasts. The model projections considering the OCV campaign and the subsequent analysis on the vaccination coverage and efficacy foresaw a strong reduction in the number of cholera cases in the vaccinated departments, with an estimation of 930 (90% CI 572-1546) and 1139 (90% CI 602-1930) averted cases in Grande Anse and Sud, respectively, under the scenario with maximum rainfall (scenario S2, see Table 1). Thus, the model suggested that the OCV campaign was indeed an effective response to mitigate the risk of a second epidemic peak in the damaged departments of Grande Anse and Sud. Nevertheless, our operational recommendations during the emergency mentioned a risk of localized outbreaks because the vaccines were not deployed homogeneously in the communes of the two departments (as assumed by the model), with pockets of susceptibility potentially remaining within vaccinated areas. Our results also suggested a limited impact of the OCV campaign in the rest of the country, implying that other intervention strategies would be required towards a global cholera control in Haiti. The latters should in fact include generalized improvements in the access to safe drinking water, and in sanitation and hygiene conditions.

We re-analyzed the second projection to estimate the vaccine efficacy and coverage, two factors that can affect the impact of the vaccine campaign. Recent estimates of the efficacy of a single-dose of OCV available in the literature include *η* = 0.40 [37], *η* = 0.63 [37], and *η* = 0.87 [12]. Regarding coverage of the target population, having all doses deployed might not lead to 100% administrative coverage. This issue has been reported in the past in Haiti [38]. Causes are wrong denominators (effective population size in the commune), vaccine spill-over (people traveling to get vaccination from surrounding areas), and population displacements modifying the effective coverage over time. Our results (Fig 6) showed that projections assuming a combination of large efficacy and coverage best matched available data, thus suggesting a great success of the vaccination campaign. This has also been proved by the low number of cholera cases recorded during 2017 in the vaccinated departments: almost 200 suspected cases in Grande Anse and 400 in cases in Sud from January to September 2017 (source: MSPP).

While numerical models may provide objective and valuable information on an ongoing epidemic, a number of model assumptions and limitations exist that should be considered when attempting to translate model outputs into generalized operational recommendations (see S1 Appendix). These include: the human mobility model; the use of deterministic equations to describe a relative small number of cases; issues on model calibration; and the accuracy of reported data. In particular, the description of human mobility by a gravity model does not reflect the actual population displacement occurred after the hurricane. Mobile phone records should be accessible in near real-time to improve the description of mobility fluxes [39]. Other environmental factors, as well as the differential access to safe drinking water, should be considered in future developments of the model. For example, Khan et al. [1] highlighted that the changes in the environmental conditions caused by the passage of the Hurricane Matthew, i.e. air temperature and precipitation anomalies and/or damage to sanitation infrastructures, are associated with a generalized spread of the environmental establishment of the pathogen, causing a higher cholera risk for the southwestern regions. Also, rainfall forecasts carried out up to a month in advance were instrumental to the proposed scenarios. However, they might not be reliable enough to support further refinements of the current model, and proper forecast errors should be considered to improve the estimation of model uncertainty. Finally, while one of our focuses was on OCV, other interventions took place during the time, which may have lowered the risk of cholera, including water, sanitation and hygiene behaviour change programs. The DA procedure indirectly takes into account these interventions by adjusting the model parameters and states. However, if non-OCV interventions, including behaviour change, significantly changed during the period after vaccination, our estimates of impact may be biased in either direction.

The collaboration among the modelers at EPFL and the epidemiologists of Epicentre-MSF was crucial for the outcome of this work: on one side modelers had timely access to data that were not yet available online (e.g. weekly recorder commune level data and vaccination details), and also access to a better understanding about data collection procedures and its limitations. This granted the possibility to further improve the model for applications in near real-time, here performed for the first time. On the other side, epidemiologists could rely on the results of a spatially-explicit model that had already been tested for the Haitian cholera epidemic started in 2010, useful in particular to fix with hindsight the total number of local susceptibles at the arrival of Matthew (*S*_*i*_(0) for every commune *i*, the initial condition of the model simulations). Epidemiologists expertise was key to translate model outputs into operational recommendations, in particular to convey the probabilistic nature of the model forecasts to support decision making. To achieve this goal, model results together with the reported cases, were displayed on an online dashboard developed by Epicentre, thus improving the monitoring system of the cholera crisis after Matthew https://epicentre-msf.shinyapps.io/haiti-2016-cholera/. The dashboard, still operational as of March 2018, was internally used at MSF and shared with the Global Task Force for Cholera Control and represented one of the easiest ways to share information among partners involved in the response. Such sharing of information is fundamental to reach a common understanding of the epidemiological situation and to support the decision-making process. Data sharing could possibly further become more fluid and timely for future developments, such as mobile phone applications and automated procedures to upload the data from the surveillance network.

In conclusion, our main results may be summarized as follows:

- Spatially-explicit mathematical modeling of epidemic cholera proves an increasingly useful tool to mainstream epidemiological practice, especially when coupled to assimilation strategies to consider the available epidemiological and environmental data. Here, the spatially-explicit SIRB model has been able to provide short term accurate predictions of the Haitian outbreak, quantifying the risk associated to each department and, possibly communes, considering connectivity as a factor to influence cholera transmission.
- The first forecasts highlighted the potential risk for a resurgence of cholera cases in the months following Hurricane Matthew, where the risk was largely modulated by rainfall. This apparent risk and other field-related assessments supported the design and the implementation of a targeted oral cholera vaccine campaign. Our second forecast highlighted that this campaign had the potential to greatly mitigate cholera morbidity until the end of 2016.
- The model predicts a significantly reduced risk of cholera after implementation of the vaccination campaign in all scenarios. As expected, the predicted impact was larger in the scenarios more favorable to cholera spread.
- Sequential assimilation of epidemiological data is fundamental to update the model in near real-time and track the epidemic dynamics. Outbreak surveillance systems are therefore key to optimize cholera modeling and control interventions. Supporting high quality surveillance and data sharing among partners is an important part of both short and long-term strategies to improve the cholera prevention and control efforts. Data sharing systems that allows near real-time modeling work should be put in place to ensure the usefulness and timeliness of the modeling outputs as an additional element supporting the decision making process.

As Haiti is unlikely to be cholera-free for years to come, refined versions of this model can continue to be used to forecast future risk and evaluate the potential impacts of different control strategies. Furthermore, the core components of this model can likely be adapted to other locations rapidly. Future efforts at near real-time modeling of cholera outbreaks can be enhanced through timely data sharing, developments in our ability to rapidly identify key mechanistic features for specific epidemics in their early phase, and robust multi-disciplinary teams able to design and calibrate appropriate models and translate their outcomes.

Hopefully, the proposed approach will pave the way for future modeling-based recommendations to inform public-health interventions in response to cholera outbreaks, helping to achieve the GTFCC’s objective of reducing of 90% the number of death due to cholera by 2030 [40], with the ultimate goal that “in the twenty-first century, no one should die from cholera” [41].

## Materials and methods

### SIRB epidemiological model with vaccination

Cholera transmission is described through a spatially-explicit SIRB epidemiological model, which has been previously applied with success to the Haitian epidemic [14, 26], and that has been adapted to the present study mainly by introducing the vaccination procedure and using a spatial discretization based on the Haitian communes rather than the hydrological watersheds. Here we describe the model used in this study, highlighting the major differences with respect to previous works (in particular [14, 26]).

**Fig 7:**
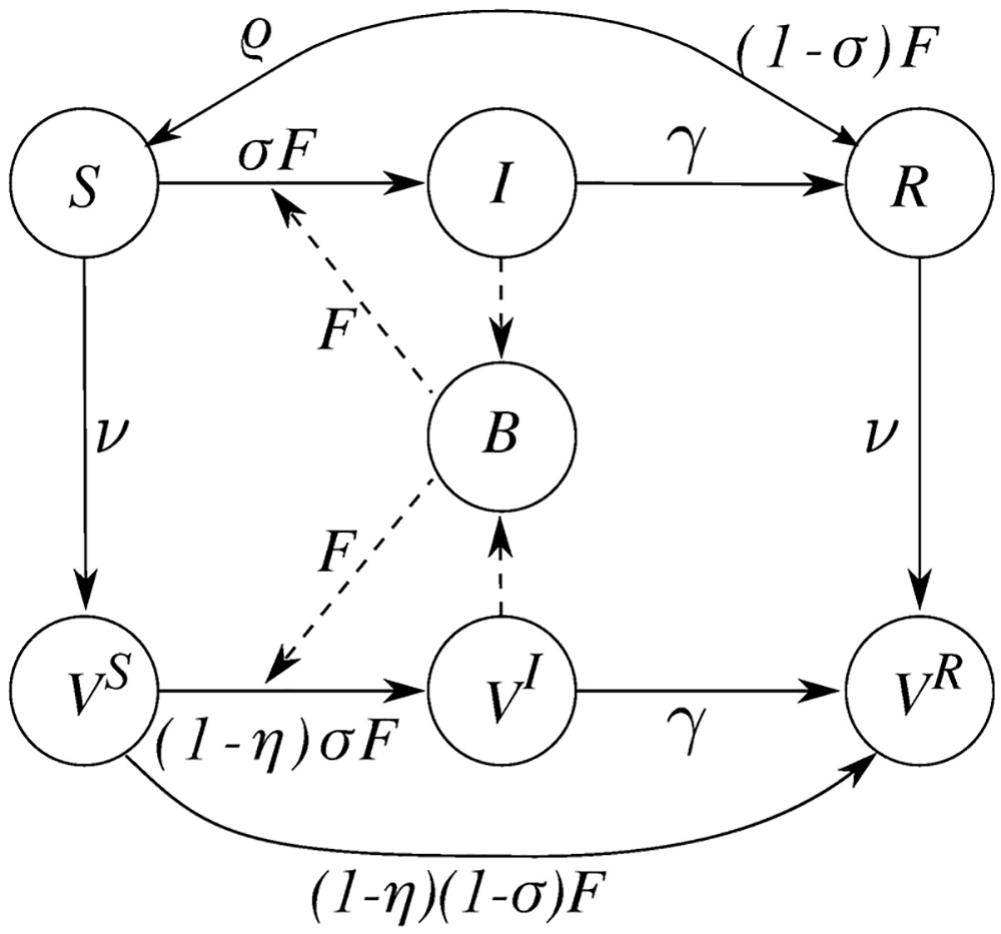
SIRBV model. Schematic representation of the SIRB model at node *i* with the three additional compartments for vaccinated individuals: vaccinated susceptible individuals (*V*^*S*^), vaccinated infected (*V*^*I*^), and vaccinated recovered (*V*^*R*^). Compartments *I* and *V*^*I*^ contribute to the local bacteria concentration (*B*). Compartment *S* is exposed to a force of infection *F*_*i*_ and enter either compartment *I* with rate *σF*_*i*_ or *R* with rate (1 − *σ*)*F*_*i*_, respectively. These probabilities are rescaled of a factor 1 − *η* for the vaccinated susceptibles.

The spatial distribution of the population is represented by *n* communities (referred to as nodes in the meta-community scheme), corresponding to the 140 Haitian communes. The number of individuals in each node *i* at time *t* are subdivided into four separate compartments, namely susceptible (*S*_*i*_), symptomatic infected (*I*_*i*_), recovered (*R*_*i*_), and vaccinated individuals (*V*_*i*_). We assume that vaccination targets individuals independently of their cholera infection history, i.e. both susceptible (*S*) and already immune individuals (*R*) are eligible with the same probability. The model assumes a linear ramp-up of vaccine uptake, meaning that daily numbers of OCV doses distributed in each community, *ν*_*i*_, is computed by equally deploying the available doses (listed in S1 Appendix) during the days of the campaign. Vaccinated individuals already immune are assumed to remain completely immune, while vaccinated susceptibles benefit from a leaky protective immunity with vaccine efficacy 0 < *η* < 1, thus reducing the susceptibility by a factor *η* (Fig 7). OCV is assumed to provide immunity one week after administration. Due to the short time horizon of the forecast, at this stage no assumption was needed for the duration of the vaccine-induced immunity i.e. vaccinated individuals remained immune for the entire duration of this study.

To take into account these assumptions, the *V*_*i*_ compartment is further subdivided into vaccinated susceptible individuals, $V_{i}^{S}$, vaccinated individuals that became infected, $V_{i}^{I}$, and vaccinated recovered who have a complete immunity, $V_{i}^{R}$. The following set of coupled ordinary differential equations describes cholera transmission dynamics:

$$\frac{dI_{i}}{dt}=\sigma F_{i}\left(t\right)S_{i}-\left(\gamma +\mu +\alpha \right)I_{i}$$ (1)

$$\frac{dR_{i}}{dt}=\left(1-\sigma \right)F_{i}\left(t\right)S_{i}+\gamma I_{i}-\left(\rho +\mu +\frac{\nu_{i}\left(t\right)}{S_{i}+R_{i}}\right)R_{i}$$ (2)

$$\frac{dV_{i}^{S}}{dt}=\nu_{i}\left(t\right)\frac{S_{i}}{S_{i}+R_{i}}-\mu V_{i}^{S}$$ (3)

$$\frac{dV_{i}^{I}}{dt}=\sigma \left(1-\eta \right)F_{i}\left(t\right)V_{i}^{S}-\left(\gamma +\mu +\alpha \right)V_{i}^{I}$$ (4)

$$\frac{dV_{i}^{R}}{dt}=\nu_{i}\left(t\right)\frac{R_{i}}{S_{i}+R_{i}}+\left(1-\sigma \right)\left(1-\eta \right)F_{i}\left(t\right)V_{i}^{S}+\gamma V_{i}^{I}-\mu V_{i}^{R}$$ (5)

$$\frac{dB_{i}}{dt}=-\mu_{B}B_{i}+\frac{p}{W_{i}}[1+\phi J_{i}\left(t\right)]\left(I_{i}+V_{i}^{I}\right)\;,$$ (6)

where the population *H*_*i*_ of each node is assumed to be at demographic equilibrium, thus $S_{i}=H_{i}-I_{i}-R_{i}-V_{i}^{S}-V_{i}^{I}-V_{i}^{R}$. The term *F*_*i*_(*t*), the force of infection, represents the rate at which susceptible individuals are exposed to contaminated water, which is expressed as a function of the bacteria concentration in the local environment, *B*_*i*_:

$$F_{i}\left(t\right)=\beta_{i}[\left(1-m\right)\frac{B_{i}}{K+B_{i}}+m\sum_{j=1}^{n}Q_{ij}\frac{B_{j}}{K+B_{j}}].$$ (7)

Parameter *β* denotes the maximum exposure rate and the fraction *B*_*i*_/(*K* + *B*_*i*_) is the probability of becoming infected due to the exposure to a concentration *B*_*i*_ of *V. cholerae*, *K* being the half-saturation constant [42]. The force of infection takes into account the possible disease propagation due to human mobility, with the idea that a fraction *m* of susceptible individuals daily commutes between nodes and can thus be exposed to pathogens at the destination. A gravity model is used to compute the probability *Q*_*ij*_ that an individual living at node *i* commutes to *j* as [43]:

$$Q_{ij}=\frac{H_{j}e^{-d_{ij}/D}}{\sum_{k\neq i}^{n}H_{k}e^{-d_{ik}/D}}\;,$$ (8)

where the attractiveness of node *j* depends on its population size *H*_*j*_, while the deterrence factor is assumed to be dependent on the distance *d*_*ij*_ between the two communities via an exponential kernel with shape factor *D*.

A fraction *σ* of the infected individuals develops symptoms, thus entering class *I*_*i*_. The remaining fraction (1 − *σ*) is asymptomatic and therefore does not contribute to the disease transmission and enters the recovered compartment directly. Symptomatically infected individuals (*I* and *V*^*I*^) recover at a rate *γ* or die due to cholera or other causes at rates *α* or *μ*, respectively. As *α* is much lower than *γ* [8], it is set to zero for simplicity. Symptomatically infected individuals contribute to the environmental concentration of *V. cholerae* at a rate *p*/*W*_*i*_, where *p* is the rate at which bacteria excreted by an infected individual reach and contaminate an conceptual environmental water reservoir of volume *W*_*i*_ (assumed to be proportional to population size, i.e., *W*_*i*_ = *cH*_*i*_ as in [3]). By introducing the dimensionless bacterial concentrations $B_{i}^{*}=B_{i}/K$, it is possible to group three model parameters into a single ratio *θ* = *p*/(*cK*) [7]. Environmental *V. cholerae* are assumed to decay at a rate *μ*_*B*_. Recovered individuals lose their immunity and return to the susceptible compartment at a rate *ρ* or die at a rate *μ*.

Flooding associated to rainfall events cause a deterioration of sanitary conditions mainly through bacterial dispersal from open-air defecation sites to the local watershed. The increased risk of water-to-human transmission is here modeled by an increase of the bacterial loads injected in the water reservoir from various environmental sources driven by intense rainfall. The contamination rate *p* is assumed to be linearly dependent to the rainfall intensity *J*_*i*_(*t*), here used as a proxy of runoff volumes, via a coefficient *ϕ* [3, 44]. The weekly cholera cases to be compared with the data are computed by integrating the number of new cases in every commune, i.e. $\sigma F_{i}S_{i}+\sigma \left(1-\eta \right)F_{i}S_{i}^{V}$.

The main innovation with respect to previous versions of the model (used, e.g., in [14, 26]) is the introduction of compartments *V*^*I*^, *V*^*S*^, and *V*^*R*^, which are necessary to describe the leaky immunity provided by OCV. Moreover, the hydrological dispersal of bacteria and the mobility of infected individuals were not considered here. The main reason motivating this assumption is to seek a simplification of the processes governing the model. While the bacteria dispersal along the hydrological network, especially the Artibonite River, may have been an important transmission process at the first stages of the Haitian epidemic [2, 45], transmission due to human mobility has been more relevant in the following years [11]. For what concern the mobility of infected individuals, most of them require urgent hydration treatment at healthcare points, thus the hypothesis that a relevant fraction of infect individuals commutes among the nodes is unconvincing.

The data necessary to apply the spatially-explicit model are the population corresponding to each model node, rainfall measurements and future projections downscaled on the nodes, and information about the initial distribution of the population in each model compartment. S1 Appendix describes the data used to setup the model and the initial conditions.

### Data assimilation and calibration

Since the model is not a perfect representation of the reality, it is important to quantify the uncertainty of model predictions.

Previous calibration attempts to the Haitian epidemic [11, 26] used a Markov Chain Monte Carlo method, the so called Differential Evolution Adaptive Metropolis (DREAM), to estimate the posterior distribution of the parameters with respect the departmental level data collected in the first years of the epidemic. For example, Bertuzzo et al. [26] calibrated the model from 2010 to 2012, leaving data collected in 2013 for the validation. In particular Mari et al. [11] performed a detailed analysis on the impact of different lengths of the calibration period, highlighting that spatially connected models outperform simpler disconnected ones for short calibration windows.

Due to the long period passed from the beginning of the epidemic to the occurrence of the hurricane (almost six years, from November 2010 to October 2016), the model calibration using DREAM would be highly time consuming and might generate an over-fit of the parameters (see [14]). As an alternative, here the probability distributions of the model results and of the parameters are inferred through a sequential DA procedure. The main idea is to correct the weekly model predictions with the reported data, in order to obtain better accuracy in the subsequent forecast. Validation of the results is then performed on the quality of the short-term forecast (see S3 Appendix). A similar DA approach in epidemiological contests had been recently proposed in other real-time efforts using DA for forecasting the spread of the Ebola during the West Africa emergency [46, 47] and to predict the peak of seasonal influenza in New York with seven weeks of advance [48].

The DA scheme considered is the squared root deterministic implementation of the ensemble Kalman filter (EnKF) with the state augmentation technique for the parameter estimation [49, 50] (see S2 Appendix). The algorithm starts by sampling *N* independent random realizations of the uncertain parameters, *ϑ* = (*β*, *m*, *D*, *ρ*, *σ*, *μ*_*B*_, *θ*, *ϕ*), which are assumed to be homogeneous is space (the same value for all nodes), with a uniform and independent prior distribution. Parameter *θ* is assumed equal to 1 due to its high correlation with *β* (see [26]). *N* initial conditions of the system are computed for each sample of the parameters (S2 Appendix) and are indicated by $x^{\left(j\right)}\left(t=0\right)=\{I_{0}^{\left(j\right)},R_{0}^{\left(j\right)},V_{0}^{S,(j)},V_{0}^{I,(j)},V_{0}^{R,(j)},B_{0}^{\left(j\right)}\}$, with *j* = 1, …, *N*. The state variables associated to each realization are independently propagated in time during each epidemiological week solving (1–6) driven by the measured GPM values of rainfall and the gravity model for human mobility. This step computes the ensemble forecast $x_{t}^{f,(j)}$, which delineate the empirical probability distribution of the state variables, $p(x_{t}^{f})$. From the ensemble of state forecasts the model computes the foreseen observations, here the forecasted weekly cases in each commune, $y_{t}^{f,(j)}$.

These model outputs have to be compared with reported cholera case data, which are typically subject to measurement errors due to various reasons, such as under-reporting in some Haitian locations, or over-reporting due to false-reporting of other diarrheal cases. The probabilistic quantification of this observational uncertainty is required by the DA procedure. We assumed that the number of cases is distributed as a negative binomial probability distribution having the mean corresponding to the number of observed cases and parameter *p* assigned (*p* = 0.8). The mass density function for a negative binomial distribution *Y* reads as

P(Y=y)=(r+y-1y)qrpy

where *y* is the number of correctly counted cases after *r* failures, *p* the probability of counting correctly and *q* = 1 − *p*. The parameter *r* is assigned imposing that the expected value of *Y*, which is $\frac{qr}{p}$, is equal to observed reported cases. The observations reporting zero cases are corrected to 0.1 in order to compute a positive *r*.

At the end of every epidemiological week, the assimilation procedure compares the number of cases foreseen by the model $y_{t}^{f,(j)}$ with the newly probability distribution of the reported cases **y**_*t*_ and consequently updates the state variables $x_{t}^{f,(j)}$ and the model parameters.

This analysis is implemented with the update step of the ensemble Kalman filter (EnKF), which is particularly suited for variables with a Gaussian distribution [49, 50]. An empirical Gaussian anamorphosis transformation of the state variables and parameters is used to improve the performance of the EnKF procedure (see S2 Appendix for more details).

## Supporting information

S1 Fig Weekly incidence and precipitation. Weekly reported cholera cases at the departmental level (dots) and mean daily precipitation as measured by the GPM mission (blue) from June to December 2016. The yellow bar represents the period of the OCV campaign, while the green bar corresponds to the date of the first model projection. The red area represents a rainfall forecast computed by the CFS on October 27.
(EPS)

S2 Fig Forecasted departmental incidence—First projection. Box-plot (percentiles 0.05, 0.25, 0.5, 0.75, 0.95) of the cumulative incidence (total number of new cases divided by the population size) forecasted in each department during after the vaccination period (from November 12 to December 31) during the first projection. Results are obtained with (green) and without (red) the deployment of the OCV campaign in Grande Anse and Sud departments (yellow background) starting on the 11th of November. The cholera projections are driven by an ensemble of forecasted rainfall (S1), the maximum forecasted rainfall (S2), and the actual rainfall (S3). The circle represents the measured incidence during the same period, while asterisks are a constant projection based on the cases reported during the first four weeks after Matthew (from October 4 to October 29).
(EPS)

S1 Appendix Haitian model setup and initial conditions. (PDF)

S2 Appendix Ensemble Kalman filter and Gaussian anamorphosis transformation. (PDF)

S3 Appendix Calibration results. (PDF)

S4 Appendix Projection analysis. (PDF)

S5 Appendix Data sources. (PDF)
