# Supplementary material for: Near real-time forecasting for cholera decision making in Haiti after Hurricane Matthew
Source: PLoS Comput Biol. 2018 May 16;14(5):e1006127. doi: 10.1371/journal.pcbi.1006127 (PMC5973636; doi:10.1371/journal.pcbi.1006127)
Supplement: S1 Appendix — (PDF) [file pcbi.1006127.s003.pdf]

Quasi real-time forecasting for cholera decision making in Haiti after Hurricane Matthew

Damiano Pasetto*, Flavio Finger, Anton Camacho, Francesco Grandesso, Sandra Cohuet, Joseph Lemaitre, Andrew S. Azman, Francisco J. Luquero, Enrico Bertuzzo, Andrea Rinaldo

* damiano.pasetto@epfl.ch

S1 Appendix. Haitian model setup and initial conditions

Haitian model setup. The computational domain of the model consists of the 140 Haitian communes, each of them hosting a human community with size extracted from the website of the Panamerican Health Organization (PAHO, `http://ais.paho.org/phip/viz/ed_haiticoleracases.asp`, accessed on 21.10.2016.). The original census data had been published by the Institut Haitien de Statistique et d'Informatique (IHSI) in March 2015 (Fig S1.1). Distances d_{ij} among the centroids of each commune have been extracted from the road network provided by the OpenStreetMap contributors (available on-line at `www.openstreetmap.org`) following the same procedure used in [1].

The daily rainfall measurements driving the model up to the beginning of the forecast are computed starting from the NASA-JAXA Global Precipitation Mission (GPM_3IMERGDL_03 late run daily precipitation estimates, resolution: 0.1 degrees, available since April 2015, see `https://www.nasa.gov/mission_pages/GPM/main` for details) which is the successor of the Tropical Rainfall Measuring Mission (TRMM) used in earlier studies.

Cholera projections into the future are driven by the precipitation forecasts provided by the NOAA's Climate Forecast System (CFS operational climate forecast having resolution of 0.938 degrees in longitude and 0.246 degrees in latitude, data available on-line at `https://www.ncdc.noaa.gov`). CFS forecasts are computed daily starting at

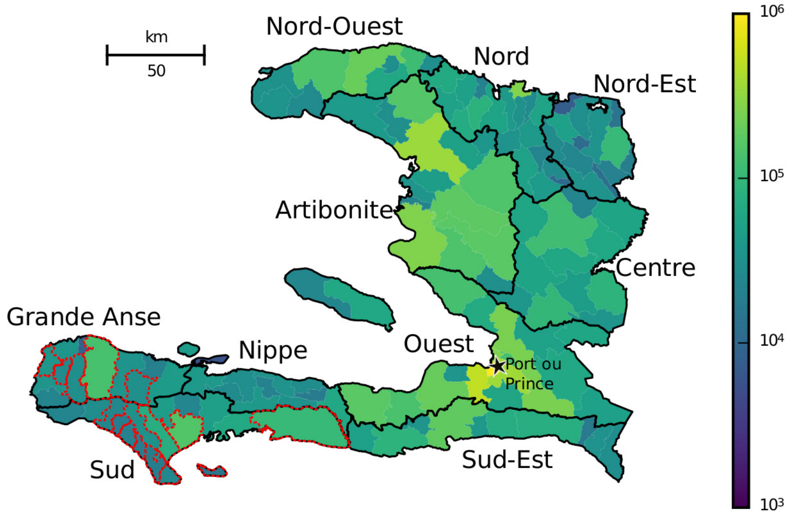

Fig S1.1. Population associated to each of the 140 communes as estimated from PAHO, `http://ais.paho.org/phip/viz/ed_haiticoleracases.asp` and communes vaccinated during the 2016 OCV campaign (red borders). The doses per commune are listed in Table S1.1

four different times (00, 06, 12, 18 UTC). For each of the four starting times the climatic data are forecasted every six hours for about nine months. We compute daily precipitation estimates for each commune by averaging the six-hours forecasts. The precipitation fields obtained from both GPM and CFS datasets are downscaled to the model nodes by interpolation on a finer regular grid of about 3 km and averaging on each commune.

Two epidemiological records are used in this study: the daily number of reported cases at the departmental level published on the website of the Haitian Ministry of Health (Ministère de la Santé Publique et de la Population MSPP, `http://mspp.gouv.ht`) since the beginning of the epidemic (October 2010), and the cases recorded at each commune during 2016 provided by MSF. The two datasets are in agreement during 2016, in the sense that the sum of the communal-level data corresponds to the departmental-level data.

We use the departmental-level data up to the end of 2015 to determine the initial condition of the model, while using the communal-level data, which is more informative of the local cholera dynamics, in the calibration and assimilation procedure during 2016.

Department	Vaccinated Commune	Estimated population
Grande Anse	Anse-d'Hainault	36401
Grande Anse	Beaumont	31580
Grande Anse	Bonbon	8610
Grande Anse	Chambellan	26459
Grande Anse	Dame-Marie	38747
Grande Anse	Jeremie	134317
Grande Anse	Les Irois	23374
Grande Anse	Moron	23374
Grande Anse	Pestel	44659
**Grande Anse**	total OCV doses	**375304**
Sud	Aquin	104216
Sud	Camp Perrin	45043
Sud	Chardonnierses	25240
Sud	Les Anglais	29891
Sud	Les Cayes	151696
Sud	Port-a-Piment	18922
Sud	Port-Salut	19098
**Sud**	total OCV doses	**394106**
**Tot**		**769410**

Table S1.1. Estimated population of the communes interested by the OCV campaign. The number of OCV doses actually used in the model during the vaccination campaign (November 11-18, 2016) is equal to the population multiplied by the vaccination coverage.

Initial conditions. In order to estimate a suitable set of parameters that describes the Haitian cholera dynamics before Matthew, the model simulations were started on February 2016, using the data collected in the previous epidemic years (from October 2010 to February 2016) as forcings in a spin-up period. This spin-up period is necessary to compute the proportion of population susceptible to cholera at the beginning of 2016, which is key to estimate the vulnerability to a new outbreak in a consistent way with respect the model parameters and the recorded cases.

Haiti's recent history being marked by a large cholera epidemic starting in 2010 and revamping every year since then, part of the population have acquired immunity that protects them from reinfection. To estimate the initial state of susceptibility of the population and the bacterial concentration in the water reservoir of each department, we used an upscaled version of equations (1-6) driven by the real force of infection,

which corresponds to the daily reported cases per department, here indicated with $\frac{dC_k}{dt}$:

$$\frac{dI_k}{dt} = \frac{dC_k}{dt} - (\gamma + \mu + \alpha)\,, I_k \tag{S1.1}$$

$$\frac{dR_k}{dt} = -(\rho + \mu)R_k + \gamma\, I_k + \frac{(1-\sigma)}{\sigma}\frac{dC_k}{dt}\,, \tag{S1.2}$$

$$\frac{dB_k}{dt} = -\mu_B B_k + \frac{p}{W_k}\left[1 + \phi J_k(t)\right](I_k)\,, \tag{S1.3}$$

where the index $k = 1, 10$ refers to the ten Haitian departments. These equations have been solved between October 20, 2010 and January 2, 2016, by assuming the initial numbers of infected and recovered to be 0 (there has been no history of cholera in Haiti in the 200 years before the start of the epidemic in 2010 [2–4]). The number of vaccinated people is set to zero, while the number of susceptibles is derived by subtracting the abundance of the other compartments from the total population, $S = H - R - I$. The results at the department level are then downscaled to the commune level proportionally to the commune population. Subsequently, a similar procedure is used at the communal level from January 2, 2016 to February 6, 2016, using as forcings the available weekly reported cases in each commune, thus ensuring that the bacterial concentration in the water reservoir at the beginning of the simulation is consistent with the data in each node of the model.

Model limitations. The deterministic equations used to simulate our model are well suited for large epidemics where demographic stochasticity is negligible. Adding stochasticity, as proposed in [5], could have been handled with the same DA approach, albeit at extra computational cost. A key spatial driver of the epidemic is based on an empirical estimation of human mobility, in this case computed by a gravity model [6]. Such assumption is consistent with the highly diffusive spread of the disease in presence of large incidence, as at the onset of the epidemics in 2010. It might not be accurate, however, for periods characterized by a low number of reported cases, when the spread might be highly stochastic. In addition, the gravity model does not account for post-hurricane specific population displacements which might have been characterized by singular characters. However, it is expected that population movements between high/low cholera incidence areas will favor the widespread propagation of the epidemic at the country-scale and thus that the gravity approach provides a reasonable

approximation. Human mobility data derived from mobile phone records have been used in Haiti and elsewhere in the context of cholera epidemics [7, 8] and could improve future approximations of human mobility if provided in close to real-time [7, 9].

A further limitation of the model might reside in the way the revamping effect of heavy rainfall is accounted for. In fact, our choice here relies on a scheme that directly models the bacterial concentration of a water reservoir (owing to washout of open-air defecation sites or to sewer overflows) that formally proved superior to all other models during the early phases of the 2010 Haiti outbreak [10] . However, after much WASH efforts especially in the southern Haitian regions hit by Matthew, it may be worth considering a comparison with models where rainfall drivers increase direct exposure probabilities rather than the environmental bacterial concentration [11].

Intrinsic limitations of the proposed methodology for predictive purposes are associated to the correct estimation of model and data uncertainty, ingredients that are key to a good performance of DA schemes. Reliable data availability in real time both on reported cases and on measured ground values of precipitation (which help constrain remote acquisition of rainfall fields) is and will remain a limiting factor, also in view of the low specificity of the reported cholera case definitions.

References

1. Pasetto D, Finger F, Rinaldo A, Bertuzzo E. Real-time projections of cholera outbreaks through data assimilation and rainfall forecasting. Advances in Water Resources. 2017;108:345–356.

2. Gaudart J, Rebaudet S, Barrais R, Boncy J, Faucher B, Piarroux M, et al. Spatio-temporal dynamics of cholera during the first year of the epidemic in Haiti. PLoS Neglected Tropical Diseases. 2013;7(4):e2145.

3. Frerichs RR, Keim PS, Barrais R, Piarroux R. Nepalese origin of cholera epidemic in Haiti. Clinical Microbiology and Infection. 2012;18(6):E158–E163.

4. Rebaudet S, et al . Cholera in coastal Africa: a systematic review of its heterogeneous environmental determinants. J Infect Dis. 2013;208:S98–S106.

5. Bertuzzo E, Finger F, Mari L, Gatto M, Rinaldo A. On the probability of extinction of the Haiti cholera epidemic. Stochastic Environmental Research and Risk Assessment. 2016;30:2043–2055. doi:10.1007/s00477-014-0906-3.

6. Erlander S, Stewart NF. The Gravity Model in Transportation Analysis – Theory and Extensions. Zeist, The Netherlands: VSP Books; 1990.

7. Bengtsson L, Gaudart J, Lu X, Moore S, Wetter E, Sallah K, et al. Using mobile phone data to predict the spatial spread of cholera. Scientific Reports. 2015;5:89–93.

8. Finger F, Genolet T, Mari L, de Magny GC, Manga NM, Rinaldo A, et al. Mobile phone data highlights the role of mass gatherings in the spreading of cholera outbreaks. Proceedings of the National Academy of Science of the United States of America. 2016;accepted.

9. Wesolowski Ae. Quantifying travel behavior for infectious disease research:A comparison of data from surveys and mobile phones. Scientific Reports. 2014;5:8923–8928.

10. Rinaldo A, Bertuzzo E, Mari L, Righetto L, Blokesch M, Gatto M, et al. Reassessment of the 2010-2011 Haiti cholera outbreak and rainfall-driven multiseason projections. Proceedings of the National Academy of Sciences of the United States of America. 2012;109(17):6602–6607.

11. Eisenberg MC, Kujbida G, Tuite AR, Fisman DN, Tien JH. Examining rainfall and cholera dynamics in Haiti using statistical and dynamic modeling approaches. Epidemics. 2013;5(4):197–207.
