# Supplementary material for: Near real-time forecasting for cholera decision making in Haiti after Hurricane Matthew
Source: PLoS Comput Biol. 2018 May 16;14(5):e1006127. doi: 10.1371/journal.pcbi.1006127 (PMC5973636; doi:10.1371/journal.pcbi.1006127)
Supplement: S2 Appendix — (PDF) [file pcbi.1006127.s004.pdf]

# Quasi real-time forecasting for cholera decision making in Haiti after Hurricane Matthew

Damiano Pasetto*, Flavio Finger, Anton Camacho, Francesco Grandesso, Sandra Cohuet, Joseph Lemaitre, Andrew S. Azman, Francisco J. Luquero, Enrico Bertuzzo, Andrea Rinaldo

* damiano.pasetto@epfl.ch

## S2 Appendix. Ensemble Kalman filter and Gaussian anamorphosis transformation

Because the state variables, the model parameters and the observations involved in the model are characterized by positive bounded distributions, a Gaussian transformation is required to correctly perform the update step of the Ensemble Kalman filter (EnKF) and back-calculate variables that are physically consistent. The idea is to transform the cumulative distribution function (CDF) of each variable into a Gaussian CDF through a nonlinear, invertible function. The transformation we used is the empirical anamorphosis function as described in [1]. For each state variable, parameters, and observations, this transformation simply build the empirical CDF associated to the ensemble, $F^{(j)} = (j - 0.5)/N$, sorting the ensemble by their rank, $j = 1, \ldots, N$. The Gaussian values corresponding to each ensemble element are computed as the quantiles associate to $F^{(j)}$, $z^{(j)} = G^{-1}(F^{(j)})$, where $G^{-1}$ is the inverse standard Gaussian CDF. Then, the update step is performed following the unbiased square root implementation of EnKF [2] applied to the transformed Gaussian variables (see Fig S2.1:

$$z^{a,(j)} = z^{f,(j)} + \mathbf{K}\left(\tilde{\mathbf{y}} - \tilde{\mathbf{y}}^{f,(j)}\right) \quad \text{(S2.1)}$$

where $\tilde{\mathbf{y}}$ and $\tilde{\mathbf{y}}^{f,(j)}$ are the Gaussian transformation associated to the real observations $\mathbf{y}$ and the model ones $\mathbf{y}^{f,(j)}$, respectively. The mass probability distribution of the

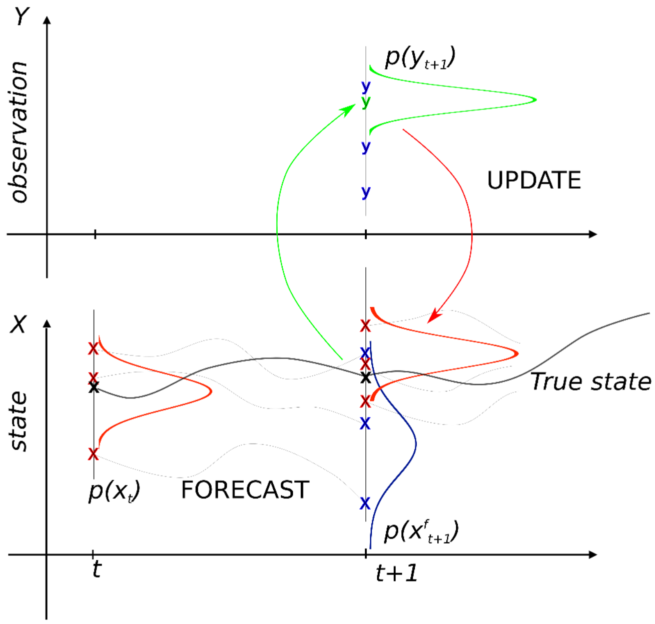


**Fig S2.1. DA scheme.** In the forecast step, the empirical pdf of the state variables (red) is propagated in time by solving the SIRB model for each realization of the ensemble (gray lines) from time $t$ to $t+1$. Then, in the update step, the empirical pdf described by the model trajectories (blue) is corrected based on the discrepancy between the measured incidence pdf ($y$ green) and the forecasted incidence ($y$ in blue), in such a way to obtain a starting point closer to the true system dynamics for the subsequent forecast.

negative binomial associated to the observations is used to compute the quantiles for the Gaussian transformation. $\mathbf{K}$ is an empirical approximation of the Kalman filter, where the correlations between forecast and observations are computed through the ensemble (for more details see, e.g., [1, 2]).

The Gaussian variables associated to $I$ ,$R$, and $B$ which are defined at the commune level, are independently updated with respect the Gaussian transformed observation of the weekly reported cases in that commune. The Gaussian transformed parameters $(m, D, \phi, \beta, \mu_B, \rho, \sigma)$, which are uniform on the domain, are updated with respect the observations upscaled to the department level, in order to capture the global trend of the epidemic and remove local noise.

The final step of the empirical anamorphosis transformation requires to back-transform the updated ensemble from the Gaussian into the original physical space, in order to obtain the updated state variables $\mathbf{x}^{a,(j)}$. This is achieved by computing the standard Gaussian CDF of $z^{a,(j)}$ and linearly interpolating them with respect to $F^{(j)}$, $j = 1, \ldots, N$. This inverse operation requires particular attention on

how to handle the tails of the Gaussian distribution, which are not directly defined in the interpolation. This step is fundamental when the observations fall outside or on the tails of the ensemble distribution, meaning that the overall ensemble would require a large correction toward the observations. However, most of the updated variables have physical constraints that should not be crossed. For example we know that $I > 0$, $R > 0$, and $I + R < H$. For this reason, the tails are here modeled by setting boundary values corresponding to the extremes of the CDF, $F^{(0)} = 0$ and $F^{(N+1)} = 1$, which correspond to $\pm\infty$ in Gaussian variables. In particular, since the number of infected individuals might be subject to strong fluctuations during an epidemiological week, we set the lower and upper bounds of $I$ in each node as $I^{(0)} = 0.5I^{(1)}$ and $I^{(N+1)} = 2I^{(N)}$. Analogous bounds are set for the bacteria concentration, which equations are linearly dependent to the number of infected individuals. The boundaries for the recovered individuals and the model parameters are computed via linear extrapolation from the ensemble values.

In addition to these constraints, to reduce the possible filter inbreeding problem on the updated parameters, which might cause the rapid underestimation of their variance and of the model uncertainty (e.g., [3, 4]), we use an adaptive inflation of the variance associate to the observation error, thus amplifying the covariances used in the computation of the Kalman gain (e.g., [3, 4]). The idea is to repeat the update step by gradually increasing the measurement error variance, here controlled by the parameter $p$ of the negative binomial distribution. $p$ is decreased until the parameter variances $\sigma_{\vartheta_k^a}$ are higher than a desired tolerance. At the $i$-th repetition of the update, we set the parameter equal to $p/c_1^i$, with $c_1 > 1$, and the update is accepted if $\sigma_{\vartheta_k^a} > c_2\sigma_{\vartheta_k^f}$ for each parameter, with $0 < c_2 < 1$. This condition controls the decrease of the parameter variances during the simulation and, thus, of the probability space explored by the ensemble. The proposed approach is justified in our application by the high uncertainty associated with the epidemiological data, whose error variance is largely unknown.

Concerning the EnKF setup, the results presented in the following are obtained with $N = 1000$, $c_1$=4, and $c_2$=0.8, and a maximum of update repetition set to 4. The condition $S_i = H_i - I_i - R_i > 0$, for $i = 1, \ldots, n$, is checked for each realization of the ensemble, and is required to accept the updated state variables. The state variables of the realizations that do not satisfy this condition are not updated.

## References


1. Schöniger A, Nowak W, Hendricks Franssen HJ. Parameter estimation by ensemble Kalman filters with transformed data: Approach and application to hydraulic tomography. Water Resources Research. 2012;48(4):W04502. doi:10.1029/2011WR010462.

2. Livings DM, Dance SL, Nichols NK. Unbiased ensemble square root filters. Physica D: Nonlinear Phenomena. 2008;237(8):1021 – 1028. doi:10.1016/j.physd.2008.01.005.

3. Anderson JL. An adaptive covariance inflation error correction algorithm for ensemble filters. Tellus A. 2007;59(2):210–224. doi:10.3402/tellusa.v59i2.14925.

4. Li H, Kalnay E, Miyoshi T. Simultaneous estimation of covariance inflation and observation errors within an ensemble Kalman filter. Quarterly Journal of the Royal Meteorological Society. 2009;135(639):523–533. doi:10.1002/qj.371.
