# Supplementary material for: Near real-time forecasting for cholera decision making in Haiti after Hurricane Matthew
Source: PLoS Comput Biol. 2018 May 16;14(5):e1006127. doi: 10.1371/journal.pcbi.1006127 (PMC5973636; doi:10.1371/journal.pcbi.1006127)
Supplement: S3 Appendix — (PDF) [file pcbi.1006127.s005.pdf]

Quasi real-time forecasting for cholera decision making in Haiti after Hurricane Matthew

Damiano Pasetto*, Flavio Finger, Anton Camacho, Francesco Grandesso, Sandra Cohuet, Joseph Lemaitre, Andrew S. Azman, Francisco J. Luquero, Enrico Bertuzzo, Andrea Rinaldo

* damiano.pasetto@epfl.ch

S3 Appendix. Calibration results

While some model parameters have a clear physical meaning, with defined reliable upper and lower bounds (e.g., m and σ, representing the fraction of individual commuting and the fraction of exposed individual becoming asymptomatic, can vary between zero and one), the prior controlling the maximum exposure rate β, the bacteria mortality μ_B and the rainfall coefficient ϕ must be assigned. In [1] a Markov Chain Monte Carlo (MCMC) approach was adopted to initialize the parameter distribution before the DA procedure. Due to the computational cost of MCMC and its underestimation of the parameter uncertainty [1], here such step is avoided by selecting a more informative prior (Table S3.1), using a low initial exposure β, long term loss of immunity (ρ among two and four years), and bacteria mortality rate μ_B that vary in the range 10 and 50 days.

As a qualitative measure of the performance of the projections, it is useful to understand if the system observations fall within the ensemble forecasted trajectories and in which percentiles. To this goal, the rank histogram [2] illustrates the frequencies at which the observations fall among the ranks 0-20%, 20-40%, 40-60%, and 80-100% for each department and for each forecast period (Fig. S3.1). A flat rank histogram indicates a good performance of the DA procedure, with the model uncertainty that captures the system uncertainty without bias. Our results show that the forecasts tend to overestimate the number of cases in all the departments, as the rank with the highest frequency is always 0-20%. We can also notice that only few observations fall outside

Table S3.1. Model parameters Parameter values of the Haitian cholera model with the associated units as well as upper and lower boundaries. The 50th (5th - 95th) percentiles of the posterior distribution computed with the EnKF and used in the forecasts are indicated.

Par.	Units	Prior	Posterior
μ	day^{-1}	$4.5{\cdot}10^{-5}$	
γ	day^{-1}	0.20	
α	day^{-1}	0.0	
σ	day^{-1}	1.0	
β	day^{-1}	0.01 - 0.2	0.0747 (0.0515 - 0.1103)
m	–	0.01 - 0.1	0.054 (0.040 - 0.065)
D	km	1 - 100	44.4 (21.8 - 68.5)
ρ	day^{-1}	0.0006 - 0.003	0.001 (0.0009 - 0.0012)
μ_B	day^{-1}	0.01 - 0.07	0.03 (0.02 - 0.04)
σ	day^{-1}	0.003 - 0.02	0.08 (0.06 - 0.1)
ϕ	day/mm	0.01 - 0.1	0.07 (0.05 - 0.098)

the ensemble interval suggesting that, in most cases, the ensemble spread is sufficiently high to capture the real dynamic of the system.

Table S3.2 illustrates the selected error statistics of the projections at one, two, and four weeks computed at both the departmental and communal level, from June to October 2016. We denote with $y_{i,t}$ the observation on node i, $i = 1, \ldots, m$ (m=10 for the departmental level), at the observation time t, $t = 1, \ldots, T$, and with $y_{i,t}^{f,j}$ the model predictions for realization j, $j = 1, \ldots N$, The error statistics considered are the ensemble root mean squared error (eRMSE),

$$eRMSE = \sqrt{\frac{\sum_{j,i,t}\left(y_{i,t} - y_{i,t}^{f,j}\right)^2}{NTm}} \tag{S3.1}$$

which considers the global behavior of the whole ensemble, and the RMSE of the ensemble mean, mRMSE:

$$mRMSE = \sqrt{\frac{\sum_{i,t}\left(y_{i,t} - \bar{y}_{i,t}^{f}\right)^2}{Tm}} \tag{S3.2}$$

which measures the error of the ensemble mean, $\bar{y}_{i,t}^{f} = \frac{1}{N}\sum_j y_{i,t}^{f,j}$. Finally, the ensemble spread measures the average distance of the ensemble from its mean value:

$$SP = \sqrt{\frac{\sum_{j,i,t}\left(\bar{y}_{i,t}^{f} - y_{i,t}^{f,j}\right)^2}{NTm}} \tag{S3.3}$$

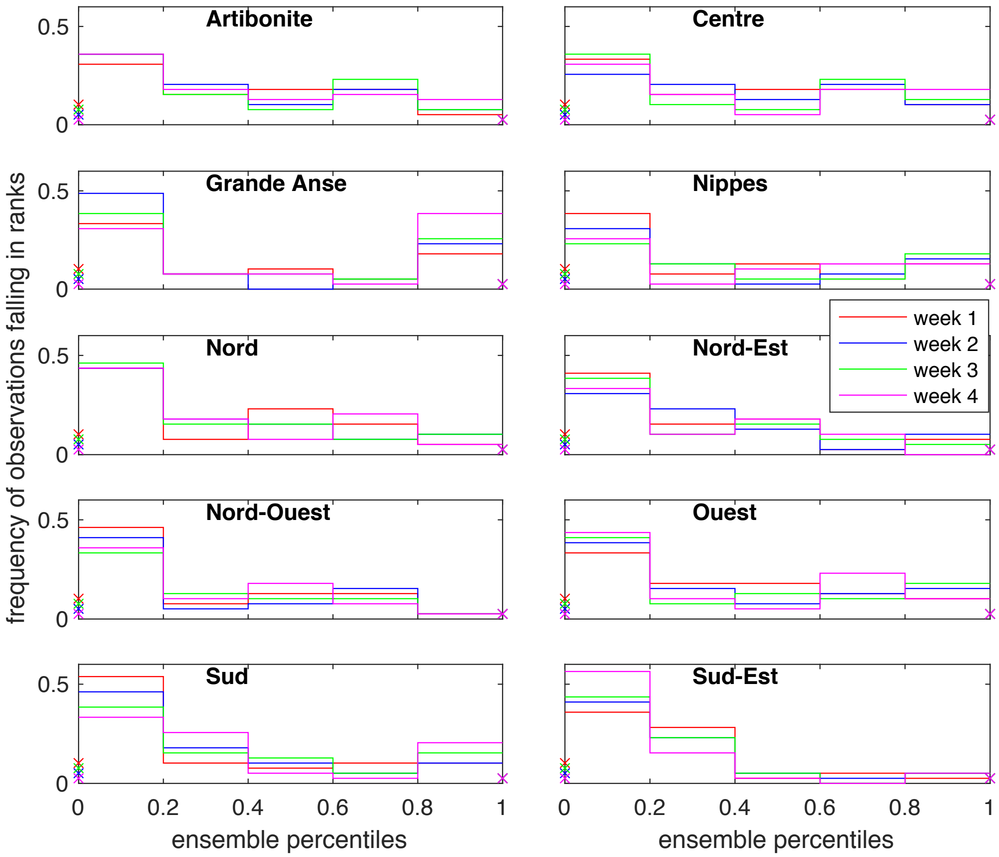

Fig S3.1. Rank histogram Rank histogram highlighting the frequency of the ensemble percentiles (ranks 0-20%, 20-40%, 40-60%, 60-80%, 80-100%) associated to the observed cases in each department and for forecasts at one, two, three and four weeks. The symbols at percentiles 0 and 100 correspond to the frequency of the observations falling outside the modeled ensemble. Simulation period: from February 2 to October 29, 2016.

From the results reported in Table S3.2 we can see that, as expected, the errors $eRMSE$ and $mRMSE$, increase for longer projections at both the communal and departmental level. At the same time, the ensemble spread SP increases for longer projections, helping to capture more observations in the ensemble confidence interval. The department having the largest error and spread is Ouest, where more cases are recorded. After Ouest, Grande Anse and Sud have the largest errors, mainly as a consequence of the raising of cholera cases after Hurricane Matthew, an increase that is not captured by the model forecast.

The number of cases at the departmental level resulting from the DA procedure are presented in Figs S3.2 and S3.3 for the projections at one and four weeks, respectively. The forecast and update steps of the DA procedure are shown in Fig S3.2, where every

week	Haiti	Ar	Ce	GA	Ni	No	NE	NO	Ou	Su	SE
$enRMSE$											
1	81.2	62.1	80.8	44.8	26.4	49.0	20.6	33.5	203.1	84.8	23.7
2	104.7	77.9	122.0	59.5	34.3	61.9	27.4	38.3	256.8	104.5	32.2
3	135.1	91.4	165.1	71.8	42.0	71.4	35.0	42.1	340.9	118.1	40.9
4	177.1	114.7	211.6	87.3	54.1	88.5	45.4	47.7	464.4	120.2	55.0
$mRMSE$											
1	67.9	47.0	52.8	40.2	22.2	34.9	15.3	27.7	172.7	81.1	19.4
2	82.9	55.9	78.3	55.9	27.2	43.8	19.5	30.6	205.0	99.0	25.2
3	99.8	61.0	104.5	69.4	31.5	46.6	23.3	31.4	249.1	111.8	29.8
4	118.5	73.0	133.0	85.0	37.0	52.5	28.9	31.9	300.4	112.6	35.3
SP											
1	44.6	40.5	61.1	19.7	14.3	34.3	13.8	18.7	106.7	24.7	13.5
2	63.9	54.2	93.5	20.3	20.8	43.7	19.1	23.1	154.7	33.5	20.1
3	91.0	68.1	127.7	18.4	27.8	54.1	26.1	28.0	232.7	37.9	28.0
4	131.6	88.4	164.5	19.7	39.4	71.2	34.9	35.4	354.1	42.0	42.2

Table S3.2. Ensemble spread and RMSE associated to the ensemble ($enRMSE$) and to the ensemble mean ($mRMSE$) for forecast at 1, 2, 3, and 4 weeks. Results presented for the total cases in Haiti and in each department.

week the model forecast is corrected in the direction of the reported cases. Note that this correction is particularly important to follow the epidemiological peak after Hurricane Matthew in Grande Anse and Sud, meaning that the strong precipitation of Matthew, used in the model to amplify the *V. cholerae* bacterial loads due by the washout of open air defecation sites or the sewer overflows, are insufficient to drive the model to high enough reported cases. This is a clear indicator that other factors should be included in the model when relevant damages to the sanitation infrastructures and important flooding occur. Fig S3.4 show the evolution in time of the model parameters. We can see that the parameter distribution is allowed to adapt during the simulation, however, the limitation imposed in the decrease of the ensemble variance avoids that the parameters collapse on one values.

Model projections computed four weeks ahead are depicted in Fig S3.3 and they are characterised by a much higher uncertainty than the weekly projections, with the model over-estimating the incidence in Centre, Ouest, Nord and Nord-Est during June 2017. However, note that these projections are always computed without knowing any information about the future incidence and sequentially updating the parameters in time, thus it is reasonable to expect large errors during the first months of simulation.

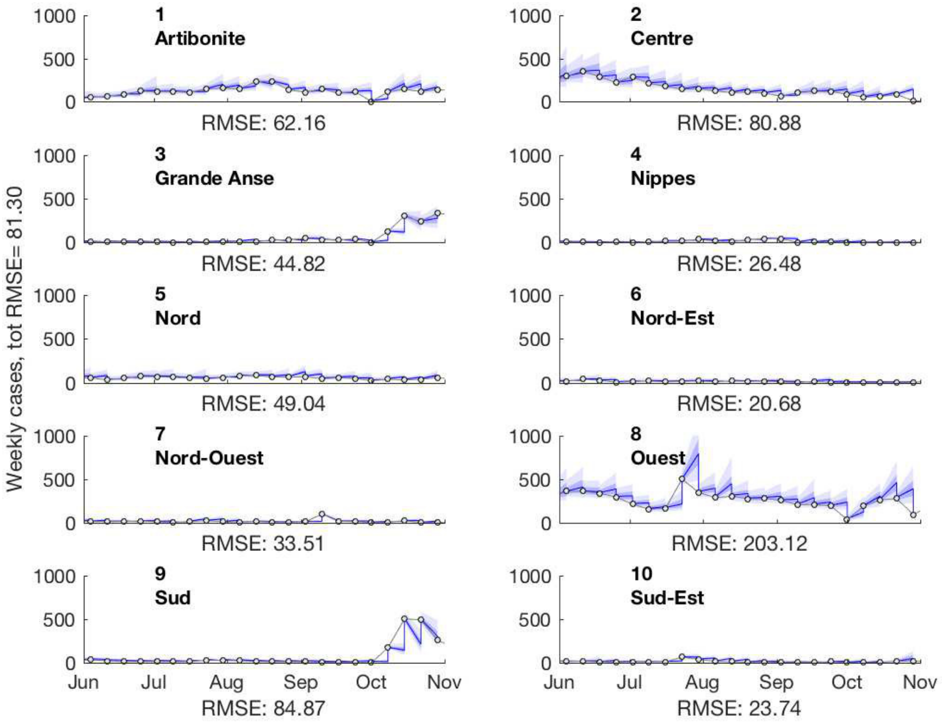

Fig S3.2. Comparison between the weekly reported cases at the departmental level and the forecast results at one week ahead. The ensemble median is the blue line, the dark blue area is the 25-75 confidence interval, while the light blue area is the 5-95 confidence interval. The vertical steps at the end of every week are the updates of the DA procedure.

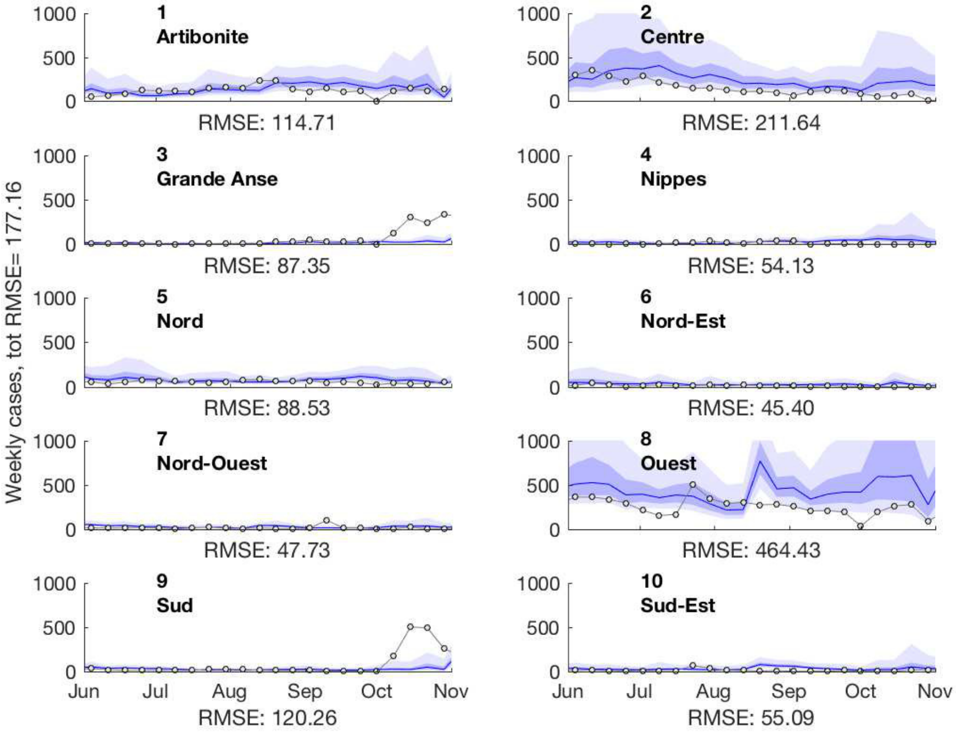

Fig S3.3. Comparison between the weekly reported cases at the departmental level and the forecast results computed four weeks ahead. The ensemble median is the blue line, the dark blue area is the 25-75 confidence interval, while the light blue area is the 5-95 confidence interval.

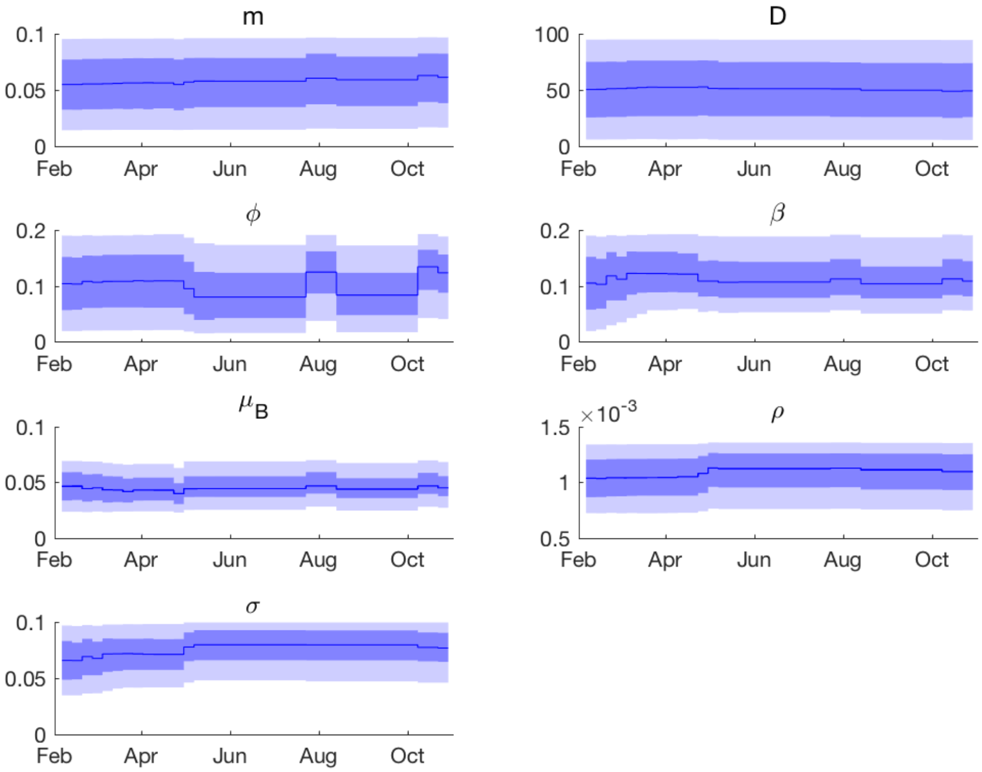

Fig S3.4. Dynamic of model parameters as updated by the DA procedure. The ensemble median is the blue line, the dark blue area is the 25-75 confidence interval, while the light blue area is the 5-95 confidence interval.

References

1. Pasetto D, Finger F, Rinaldo A, Bertuzzo E. Real-time projections of cholera outbreaks through data assimilation and rainfall forecasting. Advances in Water Resources. 2017;108:345–356.

2. Perera KC, Western AW, Robertson DE, George B, Nawarathna B. Ensemble forecasting of short-term system scale irrigation demands using real-time flow data and numerical weather predictions. Water Resources Research. 2016;52(6):4801–4822. doi:10.1002/2015WR018532.
