# Supplementary material for: Near real-time forecasting for cholera decision making in Haiti after Hurricane Matthew
Source: PLoS Comput Biol. 2018 May 16;14(5):e1006127. doi: 10.1371/journal.pcbi.1006127 (PMC5973636; doi:10.1371/journal.pcbi.1006127)
Supplement: S4 Appendix — (PDF) [file pcbi.1006127.s006.pdf]

Quasi real-time forecasting for cholera decision making in Haiti after Hurricane Matthew

Damiano Pasetto*, Flavio Finger, Anton Camacho, Francesco Grandesso, Sandra Cohuet, Joseph Lemaitre, Andrew S. Azman, Francisco J. Luquero, Enrico Bertuzzo, Andrea Rinaldo

* damiano.pasetto@epfl.ch

S4 Appendix. Projection analysis

First projection

Scenario	Department	Metric	No OCV	OCV
**S1**	**GA**	M1	0.3%	0.1%
		M2	186 (132-276)	179 (128-253)
		M3	135 (97-214)	48 (34- 70)
	**Sud**	M1	8.2%	2.5%
		M2	185 (128-338)	175 (124-278)
		M3	147 (100-283)	55 (37-101)
**S2**	**GA**	M1	5.0%	0.5%
		M2	257 (151-421)	238 (146-361)
		M3	208 (121-322)	69 (41-108)
	**Sud**	M1	70.6%	43.7%
		M2	309 (158-680)	253 (148-457)
		M3	261 (133-549)	94 (49-210)
**S3**	**GA**	M1	0.0 %	0.0%
		M2	179 (129-229)	173 (125-222)
		M3	122 (92-153)	46 (34- 58)
	**Sud**	M1	0.3 %	0.2%
		M2	173 (126-250)	169 (122-244)
		M3	127 (95-192)	51 (36- 79)

Table S4.1. Analysis first projection. (results after the vaccination, from November 12 to December 31). M1: probability of having an increase of the weekly incidence with respect to the beginning of the projection. M2: maximum weekly incidence (median and 0.05-0.95 percentiles). M3: mean weekly incidence (median and 0.05-0.95 percentiles).

Department	Weeks 40-45	Weeks 46-51 no OCV	Weeks 46-51 with OCV
Artibonite	5 (4-5)	4 (2-5)	3 (2-5)
Centre	4 (4-5)	3 (2-4)	3 (2-4)
Grande Anse	32 (28-35)	17 (12-27)	6 (4-9)
Nippes	0 (0-1)	1 (0-5)	1 (0-5)
Nord	3 (2-3)	2 (2-4)	2 (2-4)
Nord-Est	0 (0-1)	1 (0-2)	1 (0-2)
Nord-Ouest	0 (0-1)	1 (0-2)	1 (0-2)
Ouest	3 (3-3)	2 (1-4)	2 (1-4)
Sud	24 (22-27)	11 (8-22)	4 (3-8)
Sud-Est	1 (1-3)	3 (1-8)	3 (1-8)
Haiti	6 (5-7)	4 (2-6)	3 (2-4)

Table S4.2. Attack rate per 10,000 individuals, first projection, scenario S1. Ensemble median and 5-95 prediction intervals of the attack rate per 10,000 individuals in each department. The results are computed during the first six weeks of the outbreak (weeks 40 - 45, from October 3 to November 12, 2016), i.e. from the passage of the Hurricane to the beginning of the epidemics, and the following six weeks (weeks 46-51, from November 13 to December 24, 2016), where the attack rate is forecasted with and without the OCV campaign. The projection have been computed under scenario S1, i.e. considering and ensemble of rainfall forecast.

Department	Weeks 40-45	Weeks 46-51 no OCV	Weeks 46-51 with OCV
Artibonite	5 (4-5)	6 (3-9)	6 (3-9)
Centre	4 (4-5)	4 (2-8)	4 (2-7)
Grande Anse	32 (27-37)	27 (15-41)	9 (5-14)
Nippes	0 (0-1)	2 (0-14)	2 (0-14)
Nord	3 (2-3)	4 (2-8)	4 (2-7)
Nord-Est	0 (0-1)	2 (0-5)	2 (0-5)
Nord-Ouest	0 (0-1)	1 (0-5)	1 (0-5)
Ouest	3 (3-3)	4 (2-8)	4 (2-8)
Sud	24 (22-28)	20 (10-42)	7 (4-16)
Sud-Est	1 (1-3)	6 (2-20)	6 (2-20)
Haiti	6 (5-7)	6 (3-12)	4 (2-9)

Table S4.3. Attack rate per 10,000 individuals, first projection, scenario S2. Ensemble median and 5-95 prediction intervals of the attack rate per 10,000 individuals in each department. The results are computed during the first six weeks of the outbreak (weeks 40 - 45, from October 3 to November 12, 2016), i.e. from the passage of the Hurricane to the beginning of the epidemics, and the following six weeks (weeks 46-51, from November 13 to December 24, 2016), where the attack rate is forecasted with and without the OCV campaign. The projection has been computed under scenario S2, i.e. considering the precipitation forecasted having the largest total amount of rainfall.

Department	Weeks 40-45	Weeks 46-51 no OCV	Weeks 46-51 with OCV
Artibonite	5 (4-5)	3 (2-4)	3 (2-4)
Centre	4 (4-5)	3 (2-4)	2 (2-4)
Grande Anse	32 (27-36)	16 (12-20)	6 (4-7)
Nippes	0 (0-1)	1 (0-4)	1 (0-4)
Nord	3 (2-4)	4 (2-7)	4 (2-6)
Nord-Est	0 (0-1)	1 (0-4)	1 (0-4)
Nord-Ouest	0 (0-1)	1 (0-2)	1 (0-2)
Ouest	3 (3-3)	2 (1-3)	2 (1-3)
Sud	24 (22-27)	10 (7-15)	4 (4-6)
Sud-Est	1 (1-3)	2 (1-6)	2 (1-6)
Haiti	6 (5-7)	3 (2-5)	3 (2-4)

Table S4.4. Attack rate per 10,000 individuals, first projection, scenario S3. Ensemble median and 5-95 prediction intervals of the attack rate per 10,000 individuals in each department. The results are computed during the first six weeks of the outbreak (weeks 40 - 45, from October 3 to November 12, 2016), i.e. from the passage of the Hurricane to the beginning of the epidemics, and the following six weeks (weeks 46-51, from November 13 to December 24, 2016), where the attack rate is forecasted with and without the OCV campaign. The projection has been computed under scenario S3, i.e. considering the precipitation measured *a posteriori*.

Second projection

Scenario	Department	Metric	No OCV	OCV
S1	Grande Anse	M1	66.2	0.0
		M2	230 (160-406)	209 (154-287)
		M3	188 (129-308)	65 (47- 98)
	Sud	M1	64.3	0.0
		M2	262 (169-457)	225 (158-340)
		M3	218 (136-364)	73 (49-120)
S2	Grande Anse	M1	75.7	0.0
		M2	254 (165-449)	219 (156-307)
		M3	227 (146-364)	71 (49-107)
	Sud	M1	77.1	0.0
		M2	325 (174-618)	237 (164-359)
		M3	273 (149-454)	81 (51-135)
S3	Grande Anse	M1	0.1	0.0
		M2	223 (160-305)	215 (154-294)
		M3	167 (124-234)	53 (38- 74)
	Sud	M1	0.0	0.0
		M2	224 (161-342)	217 (156-331)
		M3	174 (125-263)	54 (38- 85)

Table S4.5. Analysis second projection (from November 12 to December 31). M1: probability of having an increase of the weekly incidence with respect to the beginning of the projection. M2: maximum weekly incidence (median and 0.05-0.95 percentiles). M3: mean weekly incidence (median and 0.05-0.95 percentiles).

Department	Weeks 40-45	Weeks 46-51 no OCV	Weeks 46-51 with OCV
Artibonite	4	4 (3-5)	4 (3-5)
Centre	3	2 (1-3)	1 (1-3)
Grande Anse	34	24 (17-39)	8 (6-13)
Nippes	1	5 (2-11)	5 (2-10)
Nord	2	3 (2-4)	3 (2-4)
Nord-Est	1	1 (1-3)	1 (1-3)
Nord-Ouest	0	0 (0-1)	0 (0-1)
Ouest	3	2 (2-3)	2 (2-3)
Sud	23	17 (11-28)	6 (4-9)
Sud-Est	1	2 (1-4)	2 (0-3)
Haiti	5	4 (3-7)	3 (2-4)

Table S4.6. Attack rate per 10,000 individuals, second projection, scenario S1. Ensemble median and 5-95 prediction intervals of the attack rate per 10,000 individuals in each department. The results are computed during the first six weeks of the outbreak (weeks 40 - 45, from October 3 to November 12, 2016), i.e. from the passage of the Hurricane to the beginning of the epidemics, and the following six weeks (weeks 46-51, from November 13 to December 24, 2016), where the attack rate is forecasted with and without the OCV campaign. The projection have been computed under scenario S1, i.e. considering and ensemble of rainfall forecast.

Department	Weeks 40-45	Weeks 46-51 no OCV	Weeks 46-51 with OCV
Artibonite	4	4 (3-6)	4 (3-5)
Centre	3	2 (1-3)	2 (1-3)
Grande Anse	34	29 (19-47)	9 (6-14)
Nippes	1	7 (2-14)	7 (2-13)
Nord	2	3 (2-5)	3 (2-4)
Nord-Est	1	2 (1-3)	1 (1-3)
Nord-Ouest	0	0 (0-1)	0 (0-1)
Ouest	3	2 (2-4)	2 (2-4)
Sud	23	21 (12-35)	6 (4-10)
Sud-Est	1	2 (1-4)	2 (1-4)
Haiti	5	5 (3-8)	3 (2-5)

Table S4.7. Attack rate per 10,000 individuals, second projection, scenario S2. Ensemble median and 5-95 prediction intervals of the attack rate per 10,000 individuals in each department. The results are computed during the first six weeks of the outbreak (weeks 40 - 45, from October 3 to November 12, 2016), i.e. from the passage of the Hurricane to the beginning of the epidemics, and the following six weeks (weeks 46-51, from November 13 to December 24, 2016), where the attack rate is forecasted with and without the OCV campaign. The projection has been computed under scenario S2, i.e. considering the precipitation forecasted having the largest total amount of rainfall.

Department	Weeks 40-45	Weeks 46-51 no OCV	Weeks 46-51 with OCV
Artibonite	4	4 (3-5)	3 (3-4)
Centre	3	1 (1-2)	1 (1-2)
Grande Anse	34	21 (16- 30)	7 (5-9)
Nippes	1	4 (2-8)	4 (2-8)
Nord	2	3 (2-4)	3 (2-4)
Nord-Est	1	1 (1-3)	1 (1-2)
Nord-Ouest	0	0 (0-1)	0 (0-1)
Ouest	3	2 (1-3)	2 (1-3)
Sud	23	14 (10-20)	4 (3-7)
Sud-Est	1	2 (0-3)	2 (0-3)
Haiti	5	4 (3-6)	3 (2-4)

Table S4.8. Attack rate per 10,000 individuals, second projection, scenario S2. Ensemble median and 5-95 prediction intervals of the attack rate per 10,000 individuals in each department. The results are computed during the first six weeks of the outbreak (weeks 40 - 45, from October 3 to November 12, 2016), i.e. from the passage of the Hurricane to the beginning of the epidemics, and the following six weeks (weeks 46-51, from November 13 to December 24, 2016), where the attack rate is forecasted with and without the OCV campaign. The projection has been computed under scenario S3, i.e. considering the precipitation measured *a posteriori*.

Grande Anse Commune		First projection OCV	First projection Averted	Second projection OCV	Second projection Averted
Abricots	S1	4 (1-11)	0 (0-1)	35 (19-70)	1 (0-3)
RC 41	S2	7 (2-23)	0 (0-1)	55 (27-115)	1 (0-7)
	S3	3 (1-7)	0 (0-1)	28 (18-45)	0 (0-1)
**Anse-d'Hainault**	S1	79 (47-106)	139 (84-198)	66 (39-92)	129 (68-186)
RC 55	S2	103 (54-140)	184 (100-232)	66 (39-93)	153 (77-214)
	S3	75 (46-94)	122 (77-152)	53 (32-74)	124 (70-171)
**Beaumont**	S1	0 (0-1)	1 (0-5)	0 (0-1)	1 (0-5)
RC 0	S2	1 (0-3)	3 (0-11)	0 (0-1)	2 (0-8)
	S3	0 (0-1)	1 (0-4)	0 (0-1)	1 (0-3)
**Bonbon**	S1	0 (0-1)	1 (0-3)	5 (3-7)	15 (9-26)
RC 0	S2	0 (0-2)	2 (0-6)	5 (3-7)	23 (12-41)
	S3	0 (0-1)	1 (0-2)	4 (2-5)	13 (9-19)
**Chambellan**	S1	52 (35-96)	137 (87-284)	55 (38-90)	152 (93-292)
RC 0	S2	88 (46-166)	236 (119-411)	56 (38-91)	213 (119-404)
	S3	48 (33-76)	109 (77-169)	42 (28-68)	128 (91-222)
Corail	S1	1 (0-4)	0 (0-0)	1 (0-4)	0 (0-0)
RC 0	S2	2 (0-9)	0 (0-0)	1 (0-5)	0 (0-1)
	S3	1 (0-4)	0 (0-0)	1 (0-3)	0 (0-0)
**Dame-Marie**	S1	6 (4-10)	17 (10-38)	6 (4-10)	23 (13-45)
RC 48	S2	10 (5-18)	32 (15-67)	7 (4-10)	36 (18-68)
	S3	5 (4-8)	13 (9-20)	5 (3-7)	20 (14-27)
**Jeremie**	S1	78 (57-117)	180 (123-343)	152 (102-277)	397 (248-806)
RC 376	S2	113 (70-188)	299 (172-534)	163 (107-293)	518 (299-1078)
	S3	76 (56-105)	159 (117-221)	118 (79-199)	356 (254-611)
**Les'Irois**	S1	22 (12-36)	44 (20-75)	18 (11-26)	37 (20-60)
RC 15	S2	29 (16-48)	59 (29-94)	18 (11-27)	45 (24-70)
	S3	21 (11-32)	37 (18-59)	14 (9-20)	35 (19-55)
**Moron**	S1	44 (30-57)	80 (50-115)	43 (29-52)	82 (52-105)
RC 39	S2	56 (36-75)	107 (64-142)	44 (30-52)	95 (57-124)
	S3	42 (29-50)	72 (45-88)	35 (25-41)	81 (50-98)
**Pestel**	S1	1 (0-4)	4 (1-14)	1 (0-2)	3 (1-7)
RC 0	S2	2 (1-9)	8 (2-33)	1 (0-2)	4 (1-11)
	S3	1 (0-5)	4 (1-11)	1 (0-1)	2 (1-5)
Roseaux	S1	2 (1-7)	0 (0-1)	15 (10-24)	0 (-0-1)
RC 5	S2	4 (1-15)	0 (0-1)	19 (12-30)	1 (0-2)
	S3	2 (1-6)	0 (0-0)	13 (9-19)	0 (0-0)

Table S4.9. Forecasted and averted cases per each commune of department Grande Anse. Ensemble median and 5-95 prediction intervals of the forecasted cholera cases considering the OCV campaign and the associated averted cases in each commune of department Sud for both projections. The results are presented from the beginning of the epidemics (weeks 46-51, from November 13 to December 31, 2016). RC indicates the communal reported cases during this period. Underlined communes are the communed targeted by the OCV campaign.

Sud Commune		First projection OCV	First projection Averted	Second projection OCV	Second projection Averted
**Aquin**	S1	5 (1-13)	19 (5-66)	17 (9-26)	64 (31-123)
RC 6	S2	10 (2-32)	46 (11-172)	19 (10-29)	88 (39-175)
	S3	4 (1-11)	15 (4-38)	11 (6-17)	49 (25-83)
Arniquet	S1	6 (2-17)	0 (0-2)	5 (1-12)	1 (0-2)
RC 0	S2	11 (3-41)	1 (0-2)	7 (2-19)	1 (0-4)
	S3	4 (1-10)	0 (0-1)	4 (1-8)	0 (0-1)
**Camp Perrin**	S1	10 (6-20)	34 (18-94)	7 (4-14)	26 (12-64)
RC 0	S2	19 (9-41)	74 (29-190)	8 (4-14)	39 (17-103)
	S3	10 (6-17)	28 (16-55)	5 (3-9)	21 (11-44)
Cavaillon	S1	13 (3-45)	1 (0-4)	10 (3-29)	1 (0-4)
RC 0	S2	30 (7-112)	1 (0-4)	14 (4-45)	3 (1-9)
	S3	11 (3-28)	1 (0-2)	8 (2-20)	1 (0-2)
Chantal	S1	6 (2-19)	1 (0-2)	5 (2-14)	1 (0-2)
RC 0	S2	13 (3-47)	1 (0-2)	8 (2-22)	1 (0-5)
	S3	5 (2-12)	0 (0-1)	4 (1-9)	0 (0-1)
**Chardonnieres**	S1	95 (74-128)	160 (117-231)	126 (92-238)	215 (162-304)
RC 40	S2	126 (88-171)	213 (144-273)	128 (92-240)	245 (179-327)
	S3	90 (71-114)	144 (112-173)	102 (71-203)	207 (164-306)
Coteaux	S1	5 (1-14)	0 (0-1)	4 (1-10)	0 (0-1)
RC 70	S2	9 (2-33)	0 (0-1)	5 (1-15)	1 (0-3)
	S3	4 (1-8)	0 (0-1)	3 (1-6)	0 (0-1)
Iles a Vache	S1	3 (1-10)	0 (0-1)	2 (1-6)	0 (0-1)
RC 0	S2	6 (2-25)	0 (0-1)	3 (1-9)	1 (0-2)
	S3	2 (1-5)	0 (0-0)	2 (1-4)	0 (0-0)
**Les'Anglais**	S1	50 (32-89)	128 (75-270)	47 (32-78)	128 (77-242)
RC 91	S2	83 (43-162)	223 (107-404)	49 (34-78)	175 (97-311)
	S3	45 (30-71)	100 (67-164)	34 (24-53)	105 (76-170)
**Les Cayes**	S1	61 (38-118)	191 (105-457)	138 (76-234)	461 (209-911)
RC 112	S2	112 (52-255)	411 (161-1028)	165 (79-244)	711 (247-1297)
	S3	57 (38-93)	154 (97-278)	93 (57-135)	359 (197-543)
Maniche	S1	4 (1-12)	0 (0-1)	4 (1-9)	0 (0-1)
RC 0	S2	9 (2-31)	0 (0-1)	5 (1-13)	1 (0-3)
	S3	4 (1-8)	0 (0-1)	3 (1-6)	0 (0-1)
**Port-a-Piment**	S1	43 (30-65)	89 (58-147)	42 (31-62)	92 (61-138)
RC 145	S2	63 (36-101)	136 (77-198)	44 (31-62)	113 (72-169)
	S3	42 (30-58)	78 (55-105)	32 (24-44)	82 (60-113)
**Port-Salut**	S1	4 (2-9)	11 (5-38)	1 (1-3)	4 (2-14)
RC 0	S2	7 (2-22)	25 (7-92)	1 (1-3)	7 (2-23)
	S3	3 (2-8)	8 (4-22)	1 (0-2)	3 (1-8)
Roche-a-Bateau	S1	4 (1-12)	0 (0-1)	3 (1-8)	0 (0-1)
RC 0	S2	8 (2-29)	0 (0-1)	4 (1-13)	1 (0-3)
	S3	3 (1-7)	0 (0-0)	2 (1-5)	0 (0-1)
St. Jean du Sud	S1	5 (1-15)	0 (0-2)	4 (1-11)	0 (0-1)
RC 0	S2	10 (3-38)	1 (0-2)	6 (2-17)	1 (0-3)
	S3	4 (1-8)	0 (0-1)	3 (1-7)	0 (0-1)
St. Louis du Sud	S1	14 (4-40)	1 (0-4)	11 (3-29)	1 (0-4)
RC 0	S2	29 (7-103)	1 (0-4)	15 (4-40)	3 (1-9)
	S3	11 (3-25)	1 (0-2)	9 (3-19)	1 (0-2)
Tiburon	S1	5 (1-14)	0 (0-1)	4 (1-10)	0 (0-1)
RC 91	S2	9 (2-32)	0 (0-1)	5 (2-16)	1 (0-3)
	S3	3 (1-7)	0 (0-1)	3 (1-6)	0 (0-1)
Torbeck	S1	17 (5-48)	1 (0-5)	14 (4-37)	2 (0-5)
RC 0	S2	35 (9-125)	2 (0-6)	20 (6-57)	4 (1-13)
	S3	13 (4-30)	1 (0-2)	11 (3-23)	1 (0-3)

Table S4.10. Forecasted and averted cases per each commune of department Sud. Ensemble median and 5-95 prediction intervals of the forecasted cholera cases considering the OCV campaign and the associated averted cases in each commune of department Sud for both projections. The results are presented from the beginning of the epidemics (weeks 46-51, from November 13 to December 31, 2016). RC indicates the communal reported cases during this period. Underlined communes are the communed targeted by the OCV campaign.
