# Supplementary material for: Near real-time forecasting for cholera decision making in Haiti after Hurricane Matthew
Source: PLoS Comput Biol. 2018 May 16;14(5):e1006127. doi: 10.1371/journal.pcbi.1006127 (PMC5973636; doi:10.1371/journal.pcbi.1006127)
Supplement: S5 Appendix — (PDF) [file pcbi.1006127.s007.pdf]

Quasi real-time forecasting for cholera decision making in Haiti after Hurricane Matthew

Damiano Pasetto*, Flavio Finger, Anton Camacho, Francesco Grandesso, Sandra Cohuet, Joseph Lemaitre, Andrew S. Azman, Francisco J. Luquero, Enrico Bertuzzo, Andrea Rinaldo

* damiano.pasetto@epfl.ch

S5 Appendix. Data sources

- Satellite-based daily precipitation measurements from October 2010 to March 2015: TRMM 3B42 RT Derived Daily Product, `https://disc2.gesdisc.eosdis.nasa.gov/dods/TRMM_3B42RT_Daily_7.info`
- GPM satellite-based precipitation measurements from April 2015 to December 2016: GPM level 3, IMERG late run `https://disc.gsfc.nasa.gov/datacollection/GPM_3IMERGDL_05.html`
- CFS precipitation forecasts of computed on days October 22-29 and November 05-12: CSF operational forecast, 6-hourly products `https://www.ncdc.noaa.gov/data-access/model-data/model-datasets/climate-forecast-system-version2-cfsv2`
- Population of Haitian communes: extracted from PHAO website `http://ais.paho.org/phip/viz/ed_haiticoleracases.asp`
- Road network: `https://www.openstreetmap.org`
- Departmental level cholera reported cases October 2010 - August 2016: extracted from the MSPP cholera reports, `http://mspp.gouv.ht`

- Cholera reported cases at communal level from September 2016 to December 2016): online dashboard of EPICENTRE https://epicentre-msf.shinyapps.io/haiti-2016-cholera/
